# The bromodomain protein TRIM28 controls the balance between growth and invasiveness in melanoma

**DOI:** 10.15252/embr.202254944

**Published:** 2022-11-07

**Authors:** William A Nyberg, Diego A Velasquez‐Pulgarin, Tianlin He, Maria Sjöstrand, Lucia Pellé, Ruxandra Covacu, Alexander Espinosa

**Affiliations:** ^1^ Department of Medicine, Center for Molecular Medicine, Karolinska Institutet Karolinska University Hospital Stockholm Sweden; ^2^ Department of Biomedical Engineering University of Memphis Memphis TN USA; ^3^ Department of Clinical Neuroscience, Center for Molecular Medicine, Karolinska Institutet Karolinska University Hospital Stockholm Sweden; ^4^ Present address: Department of Medicine University of California San Francisco San Francisco CA USA; ^5^ Present address: Department of Medicine, Center for Cell Engineering Memorial Sloan‐Kettering Cancer Center New York NY USA

**Keywords:** Invasiveness, JUNB, Melanoma growth, Tripartite motif‐containing 28, Cancer, Chromatin, Transcription & Genomics, Signal Transduction

## Abstract

Melanoma tumors are highly metastatic partly due to the ability of melanoma cells to transition between invasive and proliferative states. However, the mechanisms underlying this plasticity are still not fully understood. To identify new epigenetic regulators of melanoma plasticity, we combined data mining, tumor models, proximity proteomics, and CUT&RUN sequencing. We focus on the druggable family of bromodomain epigenetic readers and identify TRIM28 as a new regulator of melanoma plasticity. We find that TRIM28 promotes the expression of pro‐invasive genes and that TRIM28 controls the balance between invasiveness and growth of melanoma cells. We demonstrate that TRIM28 acts via the transcription factor JUNB that directly regulates the expression of pro‐invasive and pro‐growth genes. Mechanistically, TRIM28 controls the expression of JUNB by negatively regulating its transcriptional elongation by RNA polymerase II. In conclusion, our results demonstrate that a TRIM28–JUNB axis controls the balance between invasiveness and growth in melanoma tumors and suggest that the bromodomain protein TRIM28 could be targeted to reduce tumor spread.

## Graphical abstract

TRIM28 controls JUNB transcription that in turn induces RAS signature genes and suppresses YAP1 signature genes. TRIM28 depletion therefore leads to higher JUNB expression followed by increased melanoma growth and decreased melanoma invasiveness.

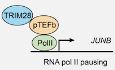

## Introduction

Cutaneous malignant melanoma originates from melanocytes in the skin and is characterized by high frequencies of somatic mutations caused by UV exposure (Alexandrov *et al*, 2013; Lawrence *et al*, 2013). Melanoma cells are highly metastatic, and melanoma metastases frequently relapse after initially successful treatment (Schachter *et al*, 2017; Wolchok *et al*, 2017). This resilience and aggressiveness of melanoma are partly caused by the plasticity of melanoma cells, enabling them to transition between distinct transcriptional signatures of invasive and proliferative states (Hoek *et al*, 2008; Roesch, 2015; Rambow *et al*, 2018; Winder & Viros, 2018; Boumahdi & de Sauvage, 2020). The ability to transition between invasive and proliferative states indicates that this occurs through reversible epigenetic mechanisms rather than by the acquisition of new mutations (Held *et al*, 2010; Hoek & Goding, 2010; Hanahan, 2022). Therefore, identifying the epigenetic mechanisms that underlie this plasticity of melanoma cells would lead to further insights into melanoma dissemination, and potentially new therapeutic strategies.

Bromodomain‐containing proteins constitute a class of epigenetic regulators of which several members (e.g., BPTF, SMARCA2, SMARCA4, PHIP, and BRD4) control the differentiation of melanocyte stem cells or contribute to the aggressiveness of melanoma cells (Saladi *et al*, 2013; Segura *et al*, 2013; Laurette *et al*, 2015). We therefore hypothesized that bromodomain proteins play an important role in the plasticity of melanoma cells. Using a combination of data mining, functional perturbations, protein interactome analysis, and *in vivo* tumor engraftment, we have identified new roles for the bromodomain protein TRIM28, and the transcription factor JUNB, in controlling melanoma growth and metastasis.

## Results & Discussion

### High expression of the bromodomain gene TRIM28 in a cluster of aggressive melanoma tumors

**Figure 1:**
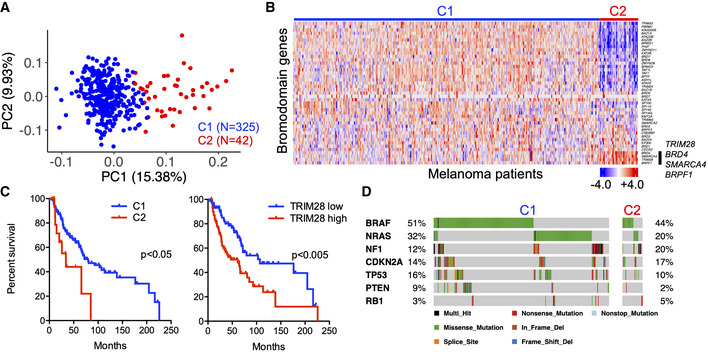
High expression of the bromodomain gene *TRIM28* in a cluster of aggressive melanoma tumors Unsupervised cluster analysis of patients with cutaneous malignant melanoma based on global gene expression (RNA‐seq) in metastases (*n* = 367). Partitioning around the medoids clustering algorithm was used (k = 2).Heatmap displaying the expression of bromodomain genes in metastases from patients with cutaneous malignant melanoma (*n* = 367). *BRD4*, *SMARCA4*, *TRIM28*, and *BRPF1* are highlighted by a black bar. Gene expression is represented by *z*‐score.Kaplan–Meier analysis of overall survival of patients with stage III melanoma in C1 (median survival 79.5 months) and C2 (median survival 25.9 months), and patients with stage III melanoma with high (median survival 61.5 months) or low (median survival 107 months) TRIM28 expression. The log‐rank test was used for statistical testing of survival data.Mutation data were downloaded from The Cancer Genome Atlas, and differences in oncogene and tumor suppressor gene mutation frequencies between the C1 and C2 clusters were analyzed with Fisher's exact test.

**Figure EV1:**
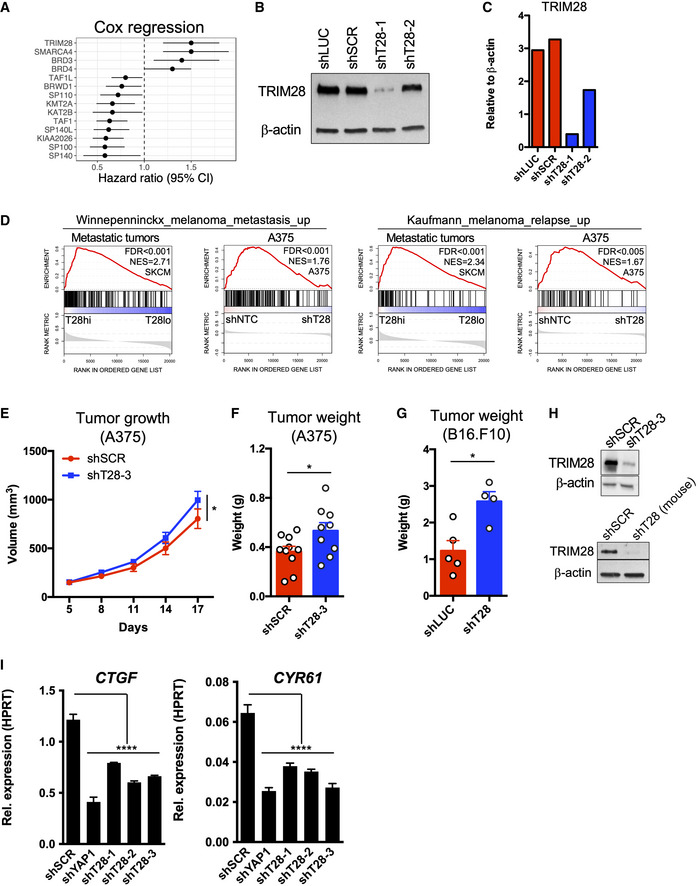
TRIM28 is associated with poor survival, increased expression of metastatic gene signatures, and the invasiveness of melanoma cells Univariate Cox regression analysis of bromodomain gene expression and overall survival in metastatic melanoma. SKCM data (TCGA) were analyzed for bromodomain gene expression in metastases (*n* = 367) and the overall survival of corresponding patients.Validation of TRIM28 knockdown using immunoblotting on cell lysates from A375 cells transduced with shLUC, shSCR, shT28‐1, or shT28‐2 lentiviruses.Densitometry (relative to β‐actin) of the immunoblot in (B).GSEA of gene signatures associated with metastasis of melanoma to distant organs (KAUFFMANN_MELANOMA_RELAPSE_UP or WINNEPENNINCKX_MELANOMA_METASTASIS_UP) in metastases (*n* = 367; *left*) with high (T28hi) or low (T28lo) TRIM28 expression, and in A375 cells (*right*) transduced with two shNTC (shSCR and shLUC) or shT28‐1 and shT28‐2 (*n* = 3 per construct). RNA‐seq data from melanoma metastases were downloaded from The Cancer Genome Atlas (TCGA).Eight‐week‐old female nude mice were injected subcutaneously with 2.5x10^6^ A375 cells transduced with shSCR or shT28‐3 lentivirus. Tumor size was measured every 3^rd^ day and tumor size was calculated using the formula V = (L × W × W)/2 (*n* = 10 mice per group). Results are expressed as mean ± SEM. Statistics were calculated using repeated measures ANOVA.Tumor weight analyzed 17 days after subcutaneous injection of cells transduced with shSCR or shT28‐3 lentivirus (*n* = 10 mice per group). Results are expressed as mean ± SEM. Statistics were calculated using the two‐sided Mann–Whitney *U*‐test.Eight‐week‐old female C57BL6/J were injected subcutaneously with 1 × 10^5^ B16.F10 cells transduced with LKO.1 lentivirus targeting Firefly luciferase (shLuc; *n* = 5) or Trim28 (shT28; *n* = 4). Tumor weight was measured after 14 days, and statistics were calculated using the two‐sided Mann–Whitney *U*‐test. Results are expressed as mean ± SEM.Validation of TRIM28 knockdown in A375 cells using shT28‐3 (top panel) and in B16.F10 using an shRNA specific for mouse *Trim28* (bottom panel).Reduced expression of the YAP1 target genes *CTGF* and *CYR61* in A375‐MA2 cells after TRIM28 knockdown. One‐way ANOVA and Dunnett's *post hoc* test were used for statistical testing. Data information: *P*‐values in (E, F, G, and I): *P < 0.05, **P < 0.01, ***P < 0.001. Source data are available online for this figure.

To identify bromodomain genes associated with aggressive melanoma, we analyzed RNA‐seq and whole‐exome sequencing data from 367 metastatic tumors together with corresponding survival data (TCGA‐SKCM). Using an unsupervised cluster analysis, we could identify two clusters of patients (Fig 1A). Cluster 2 (C2) was characterized by high expression of the bromodomain genes *TRIM28*, *SMARCA4*, *BRD4*, and *BRPF1* (Fig 1B), and survival analysis revealed that patients in C2 had significantly shorter overall survival than patients in cluster 1 (C1; Fig 1C). *TRIM28* was the bromodomain gene most strongly associated with poor overall survival (Figs 1C and EV1A). Analysis of whole‐exome sequencing data did not reveal any differences in previously described melanoma mutations between C1 and C2 (Cancer Genome Atlas Network, 2015; Fig 1D). We thus identified a subset of aggressive melanoma tumors where high expression of the bromodomain gene *TRIM28* was associated with poor survival.

**Figure 2:**
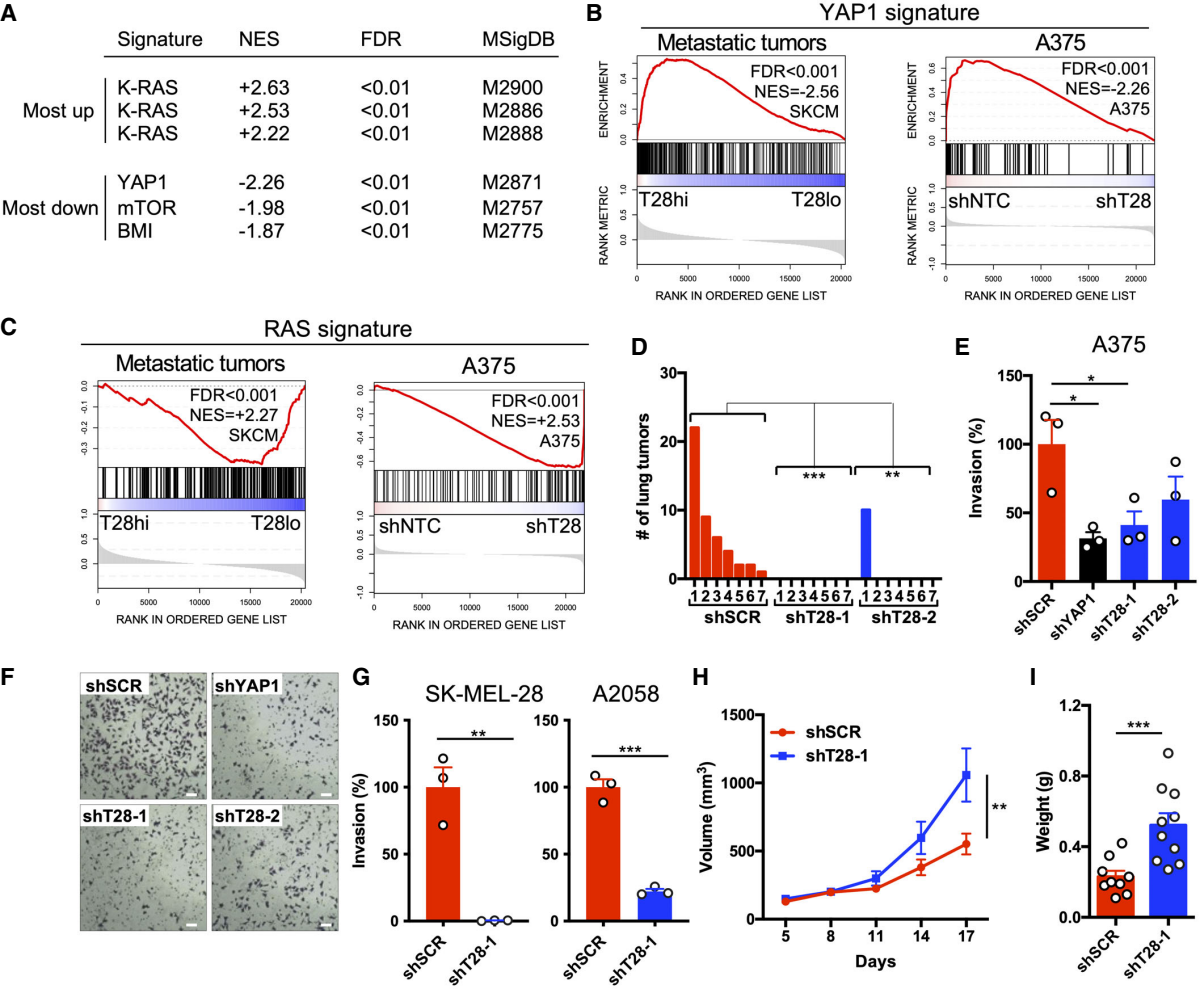
TRIM28 knockdown in melanoma cells leads to reduced invasiveness and increased tumor growth with corresponding changes in transcriptional signatures Up‐ and downregulated oncogenic gene signatures (Molecular Signatures Database, C6) in A375 cells transduced with TRIM28‐specific shRNA (shT28‐1 or shT28‐2) compared to non‐targeting control shRNA consisting of shLUC and shSCR (shNTC; *n* = 3 per construct).GSEA plot of YAP1 signature genes in TRIM28^high^ and TRIM28^low^ metastatic tumors (*n* = 367), and in A375 cells transduced with shT28‐1, shT28‐2, or shNTC (shLUC and shSCR; *n* = 3 per construct).GSEA plot of RAS signature genes in TRIM28^high^ and TRIM28^low^ metastatic tumors (*n* = 367), and in A375 cells transduced with shT28‐1, shT28‐2, or shNTC (shLUC and shSCR; *n* = 3 per construct).Number of lung tumors after intravenous injection of 1.5x10^5^ A375‐MA2 cells stably transduced with shSCR, shT28‐1, or shT28‐2 (*n* = 7 mice per shRNA). One representative experiment of two is shown. One‐way ANOVA and Tukey's *post hoc* test were used for statistical testing of tumor numbers.Matrigel invasion assays using A375 cells transduced with shSCR, shT28‐1, shT28‐2, or a YAP1‐specific shRNA (shYAP1). Results are expressed as mean ± SEM from three biological replicates (*n* = 3). One‐way ANOVA and Dunnett's *post hoc* test were used for statistical testing.Representative images from one of three Matrigel invasion assays in (E). The scale bar is 60 μm.Matrigel invasion assays using A2058 or SK‐MEL‐28 cells transduced with shSCR or shT28‐1. Results are expressed as mean ± SEM from three biological replicates (*n* = 3). Unpaired two‐sided *t*‐tests were used for statistical testing.Tumor growth after subcutaneous injection of 2.5 × 10^6^A375 cells transduced with shSCR or shT28‐1 lentivirus (*n* = 10 mice per group). Results are expressed as mean ± SEM. Repeated measures ANOVA was used for the statistical test of tumor growth.Tumor weight after the subcutaneous injection of A375 cells as shown in (H). The tumor weight was analyzed 17 days after subcutaneous injection. Results are expressed as mean ± SEM. The two‐sided Mann–Whitney *U*‐test was used for the statistical test of tumor weight. Data information: *P*‐values in (D, E, G, H, and I): *P < 0.05, **P < 0.01, ***P < 0.001.

### TRIM28 knockdown in melanoma cells leads to reduced invasiveness and increased tumor growth

TRIM28 is a multifunctional protein that mediates the repression of transposable elements, maintains epigenetic stability, and regulates transcriptional elongation by RNA polymerase II (RNAPII; Rowe *et al*, 2010; Messerschmidt *et al*, 2012; Bunch *et al*, 2014; Kauzlaric *et al*, 2020). Due to the association between high TRIM28 expression and poor survival of melanoma patients, and the previously described role of TRIM28 in cancer (Czerwinska *et al*, 2017), we hypothesized that TRIM28 controls oncogenic transcriptional signatures in melanoma cells. To test this, we first transduced A375 melanoma cells with lentivirus expressing two short‐hairpin RNA (shRNA) against TRIM28 (shT28‐1 or shT28‐2), or two non‐targeting control shRNAs (shLUC or shSCR). Knockdown efficiency was verified using immunoblotting (Fig EV1B and C), followed by global gene expression profiling. To identify changes in oncogenic transcriptional signatures after TRIM28 knockdown, we performed gene set enrichment analysis (GSEA) and found that the oncogenic YAP1 signature was the most repressed transcriptional signature after TRIM28 knockdown, while the oncogenic KRAS signature (RAS signature) was the most upregulated transcriptional signature (Fig 2A). We then asked if TRIM28 expression also was associated with a similar shift in transcriptional signatures in metastatic tumors from melanoma patients. To determine this, we analyzed RNA‐seq data from 367 metastatic tumors from melanoma patients and indeed found that low TRIM28 expression was associated with a weak YAP1 transcriptional signature (Zanconato *et al*, 2015) and a strong RAS signature (Fig 2B and C). Furthermore, metastatic transcriptional signatures were reduced both in tumors with low TRIM28 levels and in A375 cells after TRIM28 knockdown (Fig EV1D). In all, these results demonstrate that reduced TRIM28 levels in melanoma lead to reduced YAP1 and metastatic transcriptional signatures and an increased RAS signature.

YAP1 activation promotes migration and invasiveness of melanoma cells (Lamar *et al*, 2012; Nallet‐Staub *et al*, 2014; Zhang *et al*, 2020), while RAS signaling promotes melanoma growth via induction of, e.g., CXCL8 (IL‐8; Sparmann & Bar‐Sagi, 2004; Pylayeva‐Gupta *et al*, 2011). Therefore, the reduced YAP1 signature after TRIM28 knockdown suggested that TRIM28 is necessary for the invasive potential of melanoma cells. Conversely, the increased RAS signature after TRIM28 knockdown suggested that TRIM28 is a negative regulator of melanoma growth. We thus hypothesized that TRIM28 simultaneously promotes melanoma invasiveness and suppresses melanoma growth. To test this, we first performed a lung colonization experiment and injected nude mice intravenously with A375‐MA2 cells stably expressing shT28‐1, shT28‐2, or scrambled control shRNA. We first verified that TRIM28 knockdown in A375‐MA2 cells reduced the expression of YAP1 target genes (Fig EV1I). Eight weeks after injection, all mice injected with scrambled control A375‐MA2 cells had lung tumors, while only one mouse injected with shT28‐1 or shT28‐2 A375‐MA2 cells had lung tumors (Fig 2D). Next, to determine the role of TRIM28 in melanoma invasiveness, we performed Matrigel invasion assays after knockdown of TRIM28 and found that TRIM28 was necessary for the invasiveness of A375, SK‐MEL‐28, and A2058 cells (Fig 2E–G). After establishing the importance of TRIM28 for lung colonization and invasiveness, we then asked if TRIM28 also plays a role in restricting melanoma growth. To answer this, we first did subcutaneous injections of A375 cells after the TRIM28 knockdown. Indeed, the knockdown of TRIM28 in melanoma cells with two different shRNAs led to more rapid tumor growth compared to scrambled control cells (Figs 2H and I, and EV1E–H). Interestingly, we did not observe increased *in vitro* proliferation of A375 cells after TRIM28 knockdown (data not shown), suggesting that the increased growth phenotype only occurs in tumors *in vivo*. Taken together, these results demonstrate that TRIM28 knockdown induces a shift from invasiveness to tumor growth in melanoma.

### TRIM28 negatively regulates the transcriptional elongation and expression of JUNB

**Figure 3:**
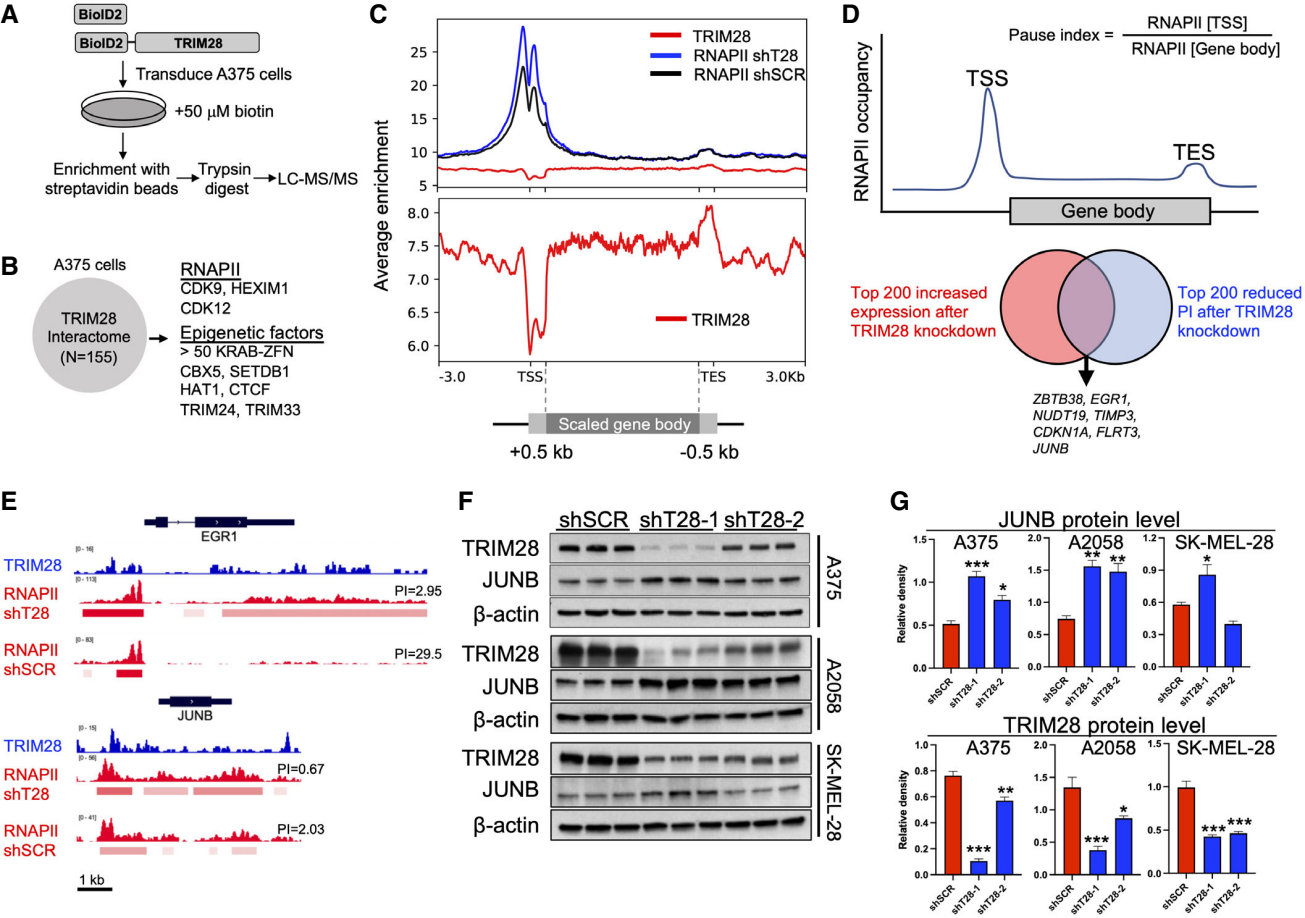
TRIM28 controls the transcriptional elongation and expression of JUNB A375 cells were transduced with pBABE‐BioID2‐TRIM28 or pBABE‐BioID2 retrovirus. Transduced cells were cultured in a complete DMEM medium containing biotin for 24 h, followed by enrichment of biotinylated proteins using streptavidin beads, and identification of interactors with LC–MS/MS.Identified TRIM28 interactome in A375 cells.Metagene profiles after CUT&RUN sequencing of TRIM28 in untransduced A375 cells and RNAPII in A375 cells transduced with shT28‐1 or shSCR lentivirus. The lower panel is focused on the TRIM28 metagene profile to clearly depict its binding profile around TSS.Global pause indexes were calculated based on RNAPII occupancy at the TSS and gene body.TRIM28 and RNAPII occupancy at *EGR1* and *JUNB* in A375 as determined by CUT&RUN sequencing (PI = length‐normalized pause index).Immunoblots against TRIM28 and JUNB in A375, A2058, and SK‐MEL‐28 cells transduced with shSCR, shT28‐1, or shT28‐2 lentivirus. Results are expressed as mean ± SEM from three biological replicates (*n* = 3).Densitometry of JUNB and TRIM28 protein levels (relative to β‐actin) in A375, A2058, and SK‐MEL‐28 melanoma cells based on the immunoblots in F. One‐way ANOVA with Dunnett's *post hoc* test was used for statistical testing. Results are expressed as mean ± SEM. *P < 0.05, **P < 0.01, ***P < 0.001. Source data are available online for this figure.

**Figure EV2:**
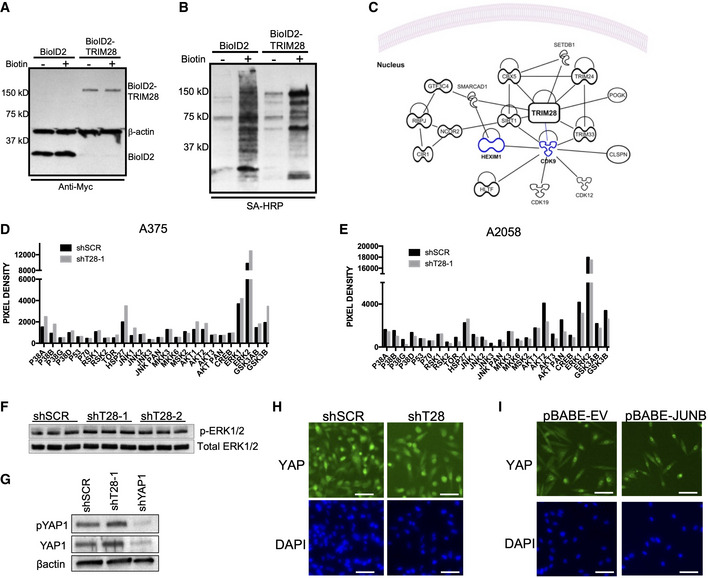
TRIM28 interacts with epigenetic regulators and factors regulating transcriptional elongation and does not directly affect ERK or YAP signaling A375 cells were transduced with pBABE‐BioID2‐TRIM28 or pBABE‐BioID2 followed by selection with 1 μg/ml puromycin. Expression of BioID2 and BioID2‐TRIM28 in cell lysates was verified with immunoblotting using an anti‐Myc antibody.A375 cells were transduced with pBABE‐BioID2‐TRIM28 or pBABE‐BioID2. Transduced cells were then cultured in the presence of 50 μM biotin for 20 h prior to lysis and then bound to Dynabeads MyOne Streptavidin C1 magnetic beads overnight. After the enrichment of biotinylated proteins, they were detected using streptavidin–HRP.Protein–protein interaction network based on identified TRIM28 interactors was analyzed by ingenuity pathway analysis (IPA). Displayed interactors are based on experimentally validated direct and indirect interactors. KRAB‐ZFN proteins (> 50) are not displayed for increased clarity.A375 cells were transduced with shSCR or shT28‐1 lentivirus followed by selection in a medium containing 1 μg/ml puromycin cells. Cells were lysed and lysates were applied to Human Phospho‐MAPK Antibody Array.A2058 cells were transduced with shSCR or shT28‐1 lentivirus followed by selection in a medium containing 1 μg/ml puromycin cells. Cells were lysed and lysates were applied to Human Phospho‐MAPK Antibody Array.A375 cells were transduced with shSCR, shT28‐1, or shYAP1 lentivirus. After selection, 1 μg/ml puromycin cells were lysed and proteins were separated on 4–20% SDS–PAGE. Proteins were transferred to PVDF membranes and detected with anti‐ERK1/2 and anti‐phospho‐ERK1/2.A375 cells were transduced with shSCR or shT28‐1 lentivirus. After selection, 1 μg/ml puromycin cells were lysed and proteins were separated on 4–20% SDS–PAGE. Proteins were transferred to PVDF membranes and detected with anti‐phospho‐YAP1 (pYAP1), anti‐YAP1, or anti‐β‐actin antibodies.Intracellular localization of YAP1 after TRIM28 knockdown in A375 cells. Scale bar is 60 μm.Intracellular localization of YAP1 after JUNB overexpression in A375 cells. The scale bar is 60 μm. Source data are available online for this figure.

**Figure EV3:**
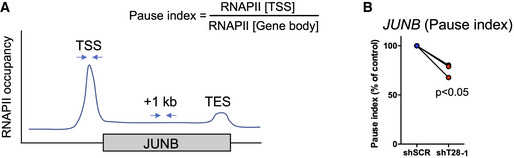
ChIP‐qPCR validation of reduced RNAPII pausing at the TSS of the JUNB gene A375 cells were transduced with shSCR or shT28‐1 lentivirus followed by selection in 1 μg/ml puromycin. After ChIP against RNAPII, qPCR was performed with primers amplifying precipitated genomic DNA from the TSS and gene body of JUNB (+1 kb).Results were normalized to corresponding input samples and three biological replicates are displayed as % pause index compared to shSCR control. The ratio *t*‐test was used for statistical testing (*n* = 3).

To elucidate how TRIM28 regulates the shift between invasiveness and tumor growth in melanoma, we first mapped the TRIM28 interactome in A375 melanoma cells (Figs 3A and EV2A–C). As expected, TRIM28 interacted with > 50 KRAB‐ZFN proteins, thus validating our proximity proteomics approach (Friedman *et al*, 1996; Kim *et al*, 1996). The TRIM28 interactome did not contain proteins involved in YAP1 or RAS signaling. Furthermore, TRIM28 knockdown did not affect the levels of phosphorylated YAP1 or ERK, or the intracellular localization of YAP1, in A375 cells, indicating that TRIM28 does not affect YAP1 or RAS signaling (Fig EV2D–I). The TRIM28 interactome did, however, include proteins known to control the transcriptional elongation by RNAPII (CDK9 and HEXIM1) and proteins involved in epigenetic regulation of gene expression (Fig 3B). We therefore reasoned that the shift from YAP1 to RAS expression signatures after TRIM28 knockdown was due to its role in RNAPII pausing or in epigenetic regulation of gene expression. To test this, we performed CUT&RUN sequencing to obtain high‐resolution data on the binding of RNAPII and TRIM28 across the genome in A375 cells. As expected, we found that metagene profiles for RNAPII showed increased RNAPII occupancy at transcriptional start sites (TSS) and transcriptional end sites (TES; Fig 3C). In contrast, the TRIM28 occupancy was reduced at TSS across the genome. Indeed, TRIM28 has previously been identified as a regulator of RNAPII transcriptional elongation whereby it controls oncogenic gene programs in cancer cells (Rowe *et al*, 2010; Messerschmidt *et al*, 2012; Bunch *et al*, 2014; Bacon *et al*, 2020; Kauzlaric *et al*, 2020). To identify genes with changes in RNAPII transcriptional elongation after TRIM28 knockdown, we calculated global RNAPII pause indexes for moderately to highly expressed genes (Fig 3D). By comparing changes in pause index and gene expression, we could identify a set of genes with increased RNAPII transcriptional elongation (decreased pause index) and increased expression after TRIM28 knockdown. These genes included *JUNB* and *EGR1* that encode transcription factors with well‐described roles in cancer cells (Fig 3E). Since JUN/FOS transcription factors have been described to control both YAP1 and RAS signaling (Zhao *et al*, 2008; Zanconato *et al*, 2015), we reasoned that increased JUNB expression might underlie the changes in expression of YAP1 and RAS transcriptional signature genes we observed after TRIM28 knockdown. We therefore first verified the increased RNAPII transcriptional elongation of *JUNB* after TRIM28 knockdown by chromatin immunoprecipitation (ChIP) and quantitative PCR (Fig EV3A and B). In addition, we also verified increased expression of *JUNB* at the protein level after TRIM28 knockdown in three melanoma cell lines (Fig 3F and G). Taken together, these results demonstrate that TRIM28 keeps the expression of *JUNB* in check by negatively regulating the transcriptional elongation of RNAPII.

**Figure 4:**
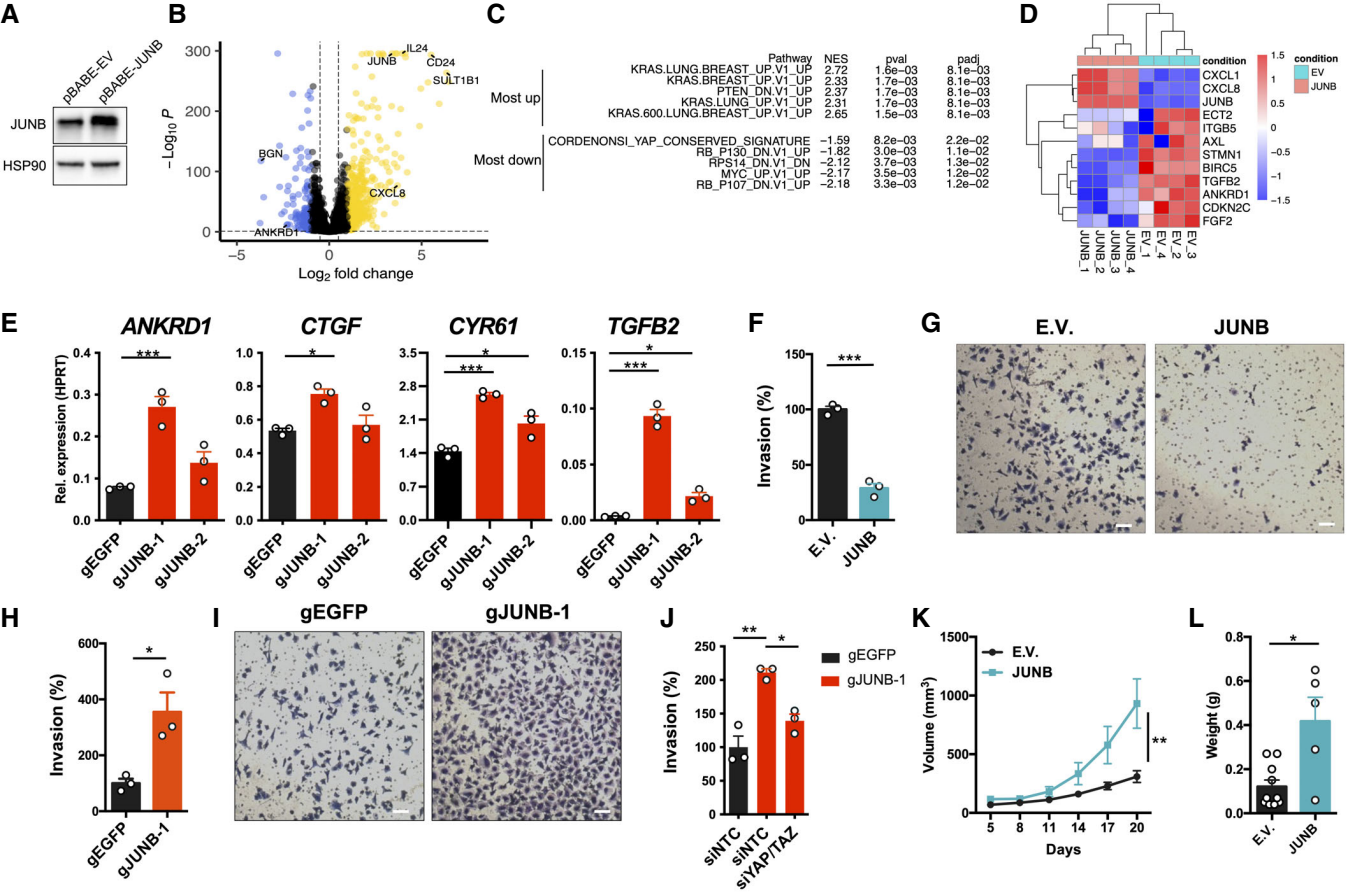
High JUNB expression leads to reduced invasiveness and increased tumor growth and corresponding changes in transcriptional signatures Immunoblotting to determine the overexpression of JUNB in A375 cells transduced with pBABE‐JUNB.Volcano plot displaying differentially expressed genes after RNA‐seq analysis of JUNB‐overexpressing A375 cells (pBABE‐JUNB) and control A375 cells transduced with empty pBABE vector (EV; *n* = 4). Genes induced after JUNB overexpression are highlighted in yellow, and genes suppressed after JUNB overexpression are highlighted in blue.GSEA analysis comparing JUNB overexpressing A375 cells to control EV cells.Heatmap displaying the induction of *CXCL1* and *CXCL8*, and suppression of YAP1 target genes, in A375 cells overexpressing JUNB.Expression of YAP1 signature genes in A375 cells after transduction with dCas9‐KRAB lentiviruses expressing gRNA targeting *JUNB* (gJUNB‐1 or gJUNB‐2) or control gRNA (gEGFP). qRT–PCR was used to determine expression levels, and results are expressed as mean ± SEM from three biological replicates (*n* = 3). One‐way ANOVA and Dunnett's *post hoc* test were used for statistical testing.Quantification of Matrigel invasion experiments with A375 cells transduced with pBABE‐JUNB (JUNB) or pBABE empty vector (EV) retrovirus. Results are expressed as mean ± SEM from three biological replicates (*n* = 3) and the two‐sided unpaired *t*‐test was used.Representative images from Matrigel invasion assays in (F). The scale bar is 60 μm.Quantification of Matrigel invasion experiments with A375 cells transduced with dCas9‐KRAB lentiviruses expressing gRNA targeting JUNB (gJUNB‐1) or control gRNA (gEGFP). Results are expressed as mean ± SEM from three biological replicates (*n* = 3) and the two‐sided unpaired *t*‐test was used.Representative images from Matrigel invasion assays in (H). The scale bar is 60 μm.Quantification of Matrigel invasion with A375 cells transduced with dCas9‐KRAB lentiviruses expressing gRNA targeting *JUNB* (gJUNB‐1) or a control gRNA (gEGFP), and then transfected with non‐targeting siRNA (siNTC) or siRNA‐targeting YAP1 (siYAP1) and TAZ (siTAZ). Results are expressed as mean ± SEM from three biological replicates (*n* = 3). One‐way ANOVA and the Tukey *post hoc* test were used for statistical testing.Tumor growth after subcutaneous injection of 2.0 × 10^6^ A375 cells transduced with pBABE‐JUNB (JUNB) or pBABE empty vector (EV) retrovirus (*n* = 10 mice per group). Results are expressed as mean ± SEM. Repeated‐measures ANOVA was used for the statistical test of tumor growth.Tumor weight after the subcutaneous injection of A375 cells as shown in (K). Tumor weight was analyzed 20 days after subcutaneous injection. Results are expressed as mean ± SEM. The two‐sided Mann–Whitney *U*‐test was used for the statistical test of tumor weight. Data information: *P*‐values in (E, F, H, J, K, and L): *P < 0.05, **P < 0.01, ***P < 0.001. Source data are available online for this figure.

### JUNB regulates the balance between invasiveness and tumor growth in melanoma

**Figure EV4:**
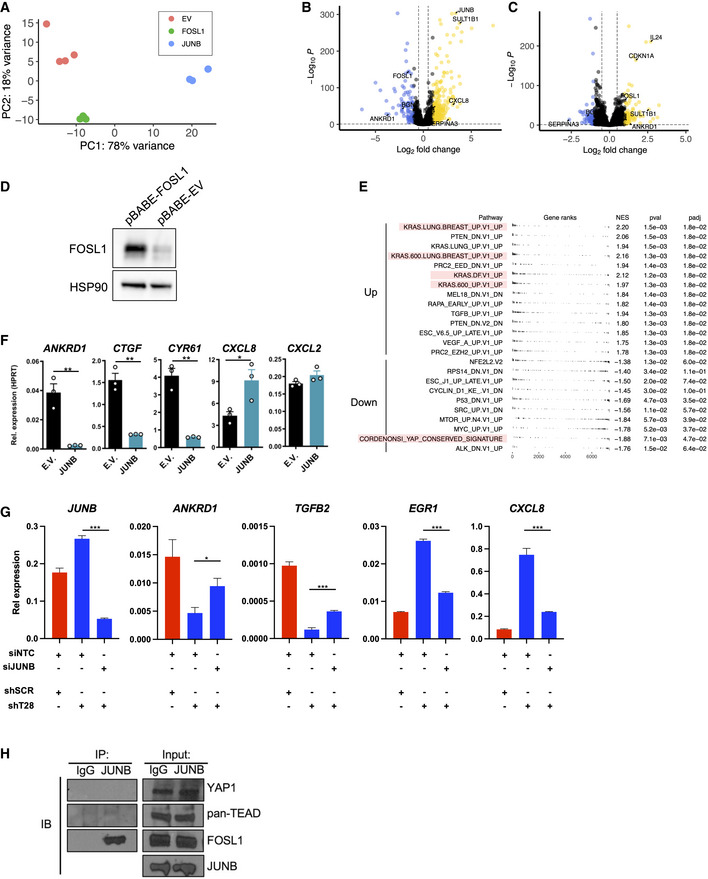
RNA‐seq of A375 cells overexpressing JUNB or FOSL1 PCA plot of the different samples in the RNA‐seq experiment (EV = empty vector; *n* = 4).Volcano plot comparing A375 cells overexpressing JUNB against A375 cells overexpressing FOSL1.Volcano plot comparing A375 cells overexpressing FOSL1 against A375 cells transduced with EV.Immunoblotting against FOSL1 after overexpression with pBABE‐FOSL1 in A375 cells. HSP90 was used as a loading control.GSEA analysis of A375 cells overexpressing JUNB compared to cells overexpressing FOSL1 (*n* = 4).Validation of JUNB overexpression effect on YAP1 target genes *ANKRD1*, *CTGF*, *CYR61*, as well as *CXCL8* and *CXCL2* in A2058 cells. qRT–PCR was used to determine expression levels and results are expressed as mean ± SEM from three biological replicates (*n* = 3). Two‐sided unpaired *t*‐tests were used for statistical testing.Rescue experiment to determine the expression of typical YAP1 and RAS signature genes after siRNA (siNTC or siJUNB) transfection of A375 cells transduced with shSCR or shT28‐1. qRT–PCR was used to determine expression levels relative to *UBC*, and the results are expressed as mean ± SEM from three biological replicates (*n* = 3). Two‐sided unpaired *t*‐tests were used for statistical testing.A375 cell lysate was used for immunoprecipitation of endogenous JUNB followed by detection by immunoblotting using anti‐YAP1, anti‐pan‐TEAD, and anti‐FOSL1. An isotype IgG antibody was used as a negative immunoprecipitation control. Data information: *P*‐values in (F and G): *P < 0.05, **P < 0.01, ***P < 0.001. Source data are available online for this figure.

**Figure EV5:**
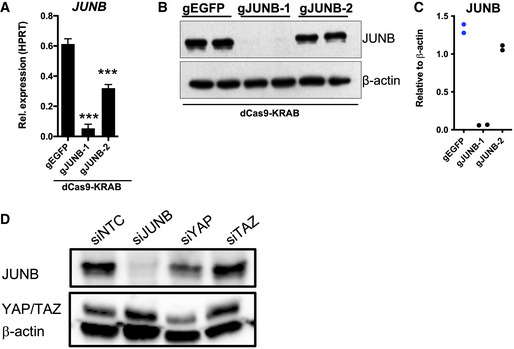
Validation of JUNB, YAP, and TAZ expression after CRISPRi and siRNA Expression of *JUNB* in A375 cells after transduction with dCas9‐KRAB lentiviruses expressing gRNA targeting *JUNB* (gJUNB‐1 or gJUNB‐2) or a control gRNA (gEGFP). qRT–PCR was used to determine *JUNB* expression levels, and the results are expressed as mean ± SEM from three biological replicates (*n* = 3). One‐way ANOVA and Dunnett's *post hoc* test were used for statistical testing. *P < 0.05, **P < 0.01, ***P < 0.001.Expression of JUNB in A375 cells after transduction with dCas9‐KRAB lentiviruses expressing gRNA targeting *JUNB* (gJUNB‐1 or gJUNB‐2) or a control gRNA (gEGFP). Immunoblotting was used to determine the protein levels of JUNB. β‐actin was used as loading control.Validation of JUNB knockdown (shown in B) by densitometry. JUNB levels are shown relative to β‐actin.Validation of siNTC‐, siJUNB‐, siYAP1‐, and siTAZ‐mediated knockdown in A375 cells. Source data are available online for this figure.

JUN and FOS transcription factors control the expression of both YAP1 and RAS signature genes (Zhao *et al*, 2008; Zanconato *et al*, 2015), and JUNB has been shown to relay a phenotypic switch underlying drug addiction in melanoma (Kong *et al*, 2017). We therefore hypothesized that the shift between YAP1 and RAS expression signatures after TRIM28 knockdown was mediated by the increased expression of JUNB. To test this, we performed RNA‐seq on A375 cells overexpressing JUNB. After performing GSEA, we observed an enrichment of oncogenic RAS signature genes in JUNB‐overexpressing cells, as well as reduced expression of YAP1 signature genes (Fig 4A–D). In contrast, FOSL1 overexpression did not suppress the YAP1 signature and did not markedly induce RAS signature genes (Fig EV4A–E). We validated the role of JUNB in suppressing YAP1 target genes (*ANKRD1*, *CTGF*, and *CYR61*) and *CXCL8* and *CXCL2* in A2058 melanoma cells (Fig EV4F). Furthermore, we used CRISPRi to knock down JUNB and found that this increased the expression of YAP1 signature genes (Fig 4E). These results indicated that increased JUNB expression strongly contributed to the effects observed after TRIM28 knockdown. To confirm the role of JUNB after TRIM28 knockdown, we performed a rescue experiment. A375 cells transduced with shT28‐1 and shSCR lentivirus were transfected with non‐targeting siRNA (siNTC) or JUNB‐targeting siRNA (siJUNB) followed by quantification of YAP1 or RAS signature genes. As hypothesized, transfection with siJUNB partially restored the expression of YAP1 and reduced the expression of RAS signature genes in TRIM28 knockdown cells (Fig EV4G). Taken together, these results demonstrated that increased JUNB expression mediated the effects of TRIM28 knockdown on the expression of YAP1 and RAS transcriptional signatures. Since JUNB controlled the expression of YAP1 target genes we asked if JUNB did this by binding directly to YAP1 or TEADs. However, after co‐immunoprecipitation of endogenous JUNB in A375 cells, we could not find any interaction with YAP1 or TEADs (Fig EV4H). Instead, we could detect the canonical interaction between JUNB and FOSL1. Since FOSL1 is a part of the YAP1 transcriptional complex and enhances the expression of YAP1 target genes (Zhao *et al*, 2008; Zanconato *et al*, 2015; Maurus *et al*, 2017), these results suggest that JUNB suppresses the expression of YAP1 target genes by sequestering FOSL1. The suppression of YAP1 signature genes by JUNB led us to hypothesize that JUNB is a negative regulator of melanoma invasiveness. Indeed, overexpression of JUNB suppressed the invasiveness of A375 cells, while knockdown of JUNB in A375 cells using CRISPRi led to increased invasiveness (Fig 4F–I). We then performed rescue experiments to test if the role of JUNB in melanoma invasiveness was dependent on YAP1. After CRISPRi against JUNB, A375 cells were transfected with non‐targeting siRNA (siNTC) or a siRNA pool against YAP1/TAZ. Seventy‐two hours after siRNA transfection, the invasiveness was determined using Matrigel invasion assays. Indeed, the knockdown of YAP1/TAZ reduced the invasiveness of A375 cells after CRISPRi of JUNB (Fig 4J), demonstrating that the invasiveness was mediated via YAP1/TAZ. We validated the CRISPRi against JUNB, and the siRNA‐mediated knockdown of JUNB, YAP1, and TAZ by immunoblotting (Fig EV5A–D). Finally, because JUNB overexpression induced the expression of RAS signature genes, we hypothesized that JUNB overexpression would cause increased tumor growth. To test this, we performed subcutaneous xenograft experiments with A375 cells, and indeed found that overexpression of JUNB led to significantly increased tumor growth (Fig 4K and L). Taken together, our data show that JUNB suppresses invasiveness and promotes tumor growth in melanoma cells.

The plasticity of cancer cells underlies their ability to metastasize and to develop drug resistance, and the transitioning between phenotypic states suggests that this occurs through reversible epigenetic mechanisms (Hoek *et al*, 2008; Held *et al*, 2010; Hanahan, 2022). TRIM28 is a multifunctional epigenetic reader that plays a critical role in maintaining self‐renewal and pluripotency of stem cells where TRIM28 is highly expressed and acts as a barrier for cellular reprogramming (Hu *et al*, 2009; Messerschmidt *et al*, 2012; Klimczak *et al*, 2017; Miles *et al*, 2017). Therefore, the high expression of TRIM28 frequently found in cancer cells might serve as a mechanism to maintain a dedifferentiated cellular phenotype (Jaworska *et al*, 2020). Here, we identified TRIM28 as a protein that controls the balance between invasiveness and growth in melanoma. Similar to our findings in melanoma cells, others have reported that TRIM28 knockdown leads to reduced invasiveness and reduced lung colonization of other cancer types (Chen *et al*, 2014; Addison *et al*, 2015). However, and in contrast to our findings, TRIM28 knockdown in non‐melanoma cancer cells also resulted in reduced tumor growth of subcutaneous and orthotopic xenografts (Addison *et al*, 2015; Fong *et al*, 2018). This difference could be due to the increased expression of pro‐growth factors CXCL2 and CXCL8 in melanoma cells after TRIM28 knockdown (Payne & Cornelius, 2002; Sparmann & Bar‐Sagi, 2004; Singh *et al*, 2009), something that was not observed after knockdown of TRIM28 in breast cancer and prostate cancer cells (Addison *et al*, 2015; Fong *et al*, 2018). TRIM28 thus controls the expression of distinctive genes in different types of cancer cells, emphasizing its pleiotropic role in the regulation of gene expression. Indeed, TRIM28 regulated the transcriptional elongation and expression of many genes in melanoma cells, and also interacted with a large number of KRAB‐ZFNs known to suppress the expression of endogenous retroviruses and transposable elements. TRIM28 might therefore act via several mechanisms to control tumor invasiveness and growth. Here, we describe that the control of JUNB expression by TRIM28 is one such mechanism. We found that the expression of JUNB is regulated at the transcriptional level by TRIM28, and that increased expression of JUNB was sufficient to repress YAP1 targets genes and simultaneously increase the expression of tumor growth factors such as CXCL8. Indeed, previous studies have demonstrated that JUN/FOS and TEAD transcription factors overlap at *cis*‐regulatory regions, thus being directly implicated in regulating the transcription of YAP1 target genes (Zanconato *et al*, 2015; Liu *et al*, 2016). Importantly, CXCL8 has emerged as a critical growth factor for melanoma, being associated with poor survival and resistance to checkpoint inhibition therapy (Sparmann & Bar‐Sagi, 2004; Schalper *et al*, 2020). In conclusion, we have uncovered a role for TRIM28 and JUNB in regulating the balance between invasiveness and growth in melanoma cells.

## Materials and Methods

### Analysis of patient data

We downloaded RNA‐seq data, mutation data, and clinical data, from patients with metastatic melanoma (TCGA‐SKCM, https://portal.gdc.cancer.gov/). To analyze RNA‐seq data from metastatic tumors of patients with survival data (*N* = 367), we first filtered gene expression (TPM) using the *genefilter* package in R using pOverA (*P* = 0.75, A = 100). We then used the *ggplot2* and *ggfortify* packages in R to perform principal component analysis. The *factoextra* package in R was used to determine the optimal numbers of clusters, and partitioning around medoids (PAM) clustering (k = 2) was performed using the *cluster* package in R. Cox regression analysis and Kaplan–Meier survival analysis were performed using the *survival* package in R and Prism 6 (GraphPad Software). For mutational analysis, we downloaded MAF files from whole‐exome sequencing of metastatic melanoma tumors (TCGA‐SKCM) and used the *maftools* package in R for mutational analysis and visualization.

### RNA sequencing and Human Transcriptome Array 2.0

A375 cells were transduced with lentiviruses expressing non‐targeting control shRNAs (shSCR or shLuc), or TRIM28‐specific shRNAs (shT28‐1 or shT28‐2), followed by selection with puromycin (1 μg/ml). Cells were harvested in cold PBS and lysed in RLT buffer (Qiagen). RNA quality and integrity control, and global gene expression with the GeneChip Human Transcriptome Array (HTA) 2.0 (Affymetrix), were performed at the Bioinformatics and Expression Analysis core facility at Karolinska Institutet. CEL files from the HTA 2.0 microarrays were preprocessed and normalized with robust multi‐array average (RMA) using the R package *oligo*. Array data are available at Gene Expression Omnibus (GSE133073). Data were log_2_ transformed and normalized to z‐scores, and heatmaps were generated using the R package *ComplexHeatmap*. For RNA sequencing, A375 cells were transduced with empty pBABE (pBABE‐EV) retrovirus, or pBABE‐expressing FOSL1 (pBABE‐FOSL1) or JUNB (pBABE‐JUNB). Total RNA was isolated using TRIzol (Invitrogen) followed by DNAseI treatment (Qiagen) and cleanup with RNeasy MinElute Cleanup kit (Qiagen). RNA quality and integrity were confirmed with an Agilent Tapestation (Agilent), and sequencing libraries were generated using the Illumina Stranded mRNA Prep kit. The indexed cDNA libraries were normalized and combined, and the pools were sequenced on the Illumina Nextseq 2000 for a P2 100 cycle sequencing run, generating 58 base paired‐end reads with dual index. Base calling and demultiplexing was performed using CASAVA software with default settings generating FASTQ files for further downstream mapping and analysis. RNA quality control, library preparation, and sequencing were performed at the Bioinformatics and Expression Analysis core facility at Karolinska Institutet. Sequences were aligned using *STAR* with default settings. Data are accessible at Gene Expression Omnibus (GSE210579). Differential gene expression was analyzed with the R package *DESeq2*, volcano plots were generated by the R package *EnhancedVolcano*, heatmaps were generated by the R package *pheatmap*, and PCA analysis was done with the *plotPCA* function in *DESeq2*.

### Gene set enrichment analysis

Expression data files (.gct), phenotype labels (.cls), and gene set files (.gmx) were uploaded to Genomspace (http://www.genomespace.org/) and analyzed using the GSEA tool in GenePattern (Subramanian *et al*, 2005). Microarray data were analyzed using log_2_‐transformed values. Normalized enrichment scores and false discovery rates were calculated as described (Subramanian *et al*, 2005). Batch GSEA was performed for oncogenic signatures (Molecular Signatures Database v6.2, 189 gene sets), while metastatic transcriptional signatures were analyzed with selected gene sets. Enrichment plots were generated using the *replotGSEA* function in R (https://github.com/PeeperLab/Rtoolbox).

For GSEA on the RNA‐seq data, we used the R package *FSGEA*.

### CUT&RUN sequencing

A375 cells (1 × 10^5^ per sample) were harvested and processed for CUT&RUN using the CUT&RUN Assay kit (Cell Signaling Technologies). Spike‐in DNA from *S. cerevisae* was added for normalization, and anti‐Rpb1 (4H8, Cell Signaling Technologies), anti‐TRIM28 (ab10484, Abcam), or IgG control (DA1E, Cell Signaling Technologies) antibodies were used for targeted digestion of chromatin. After purification of the extracted DNA, sequencing libraries were generated using NEBNext ULTRA II DNA Library Prep kit for Illumina (New England Biolabs) with indexed NEBNext Multiplex Oligos for Illumina (New England Biolabs). Size selection was performed using SPRIselect beads (Beckman Coulter) and fragment sizes were determined with an Agilent 2100 Bioanalyzer using the High Sensitivity DNA kit (Agilent). The double‐stranded DNA content of the libraries were quantified with Qubit 3.0 using the Qubit dsDNA High Sensitivity Assay kit (Thermo Fisher Scientific) followed by equimolar pooling of the indexed libraries. The pooled library was sequenced using Illumina NextSeq 550 with 2x75 bp paired‐end reads. Raw reads were analyzed with *FastQC* for quality control, and *STAR* alignment (‐alignIntronMax 1) was used to generate bam files. Peak detection was performed with *MACS2*. CUT&RUN‐sequencing signals were displayed in *Integrated Genomics Viewer* (IGV; Robinson *et al*, 2011). Data are accessible at Gene Expression Omnibus (GSE210579). Gene‐length‐normalized pause indexes were calculated for genes with moderate‐to‐high expression levels (top 70^th^ percentile) using the *getPausingIndeces* function from the BRGenomics Bioconductor package. As input, we used bedgraph files generated after CUT&RUN sequencing for RNAPII, and Granges files for TSS (−1,000 bp to +50 bp) and gene bodies (+100 bp to end of gene) from a hg38 TxDb object for known genes. We then overlapped the 200 genes with most reduced pause index after TRIM28 knockdown with the 200 genes with most induced expression after TRIM28 knockdown. The deepTools suite was used to calculate and display scaled metagene profiles with RPKM normalization to visualize the global binding of RNAPII and TRIM28. The metagene profiles were unscaled at the TSS (+0.5 kb) and transcriptional end site (TES; −0.5 kb) to better visualize the distribution of RNAPII and TRIM28 at these sites.

### Chromatin immunoprecipitation

A375 cells (3 × 10^6^) were collected and lysed for each sample. ChIP was performed using the MAGnify Chromatin Immunoprecipitation System (Invitrogen) following the manufacturer's protocol. Chromatin shearing was performed using a Bioruptor UCD‐200 sonicator running 40 cycles of 30 s on and 30 s off at high intensity (at 4°C). For each immunoprecipitation, 3 μg anti‐RNA polymerase II (ab817 Abcam) or isotype (mouse IgG, Invitrogen) antibody was added to 10 μl chromatin. Input controls were included without the addition of antibodies. Quantitative PCR was performed on purified genomic DNA with primers targeting the TSS or gene body (+1 kb) of *JUNB*. Each sample was normalized to the input control. To calculate a pause index, the occupancy of RNA polymerase II at TSS was divided by the occupancy of RNA polymerase II in the gene body.

### Cell culture

Cell lines used in this study were as follows: HEK‐293T (Espinosa laboratory stock), A375 (American Type Culture Collection), A375‐MA2 (American Type Culture Collection), SK‐MEL‐28 (American Type Culture Collection), A2508 (American Type Culture Collection), and B16‐F10 (American Type Culture Collection). All cell lines were routinely tested for mycoplasma contamination. Cells were cultured in high‐glucose DMEM (Sigma Aldrich) supplemented with fetal calf serum (10%), streptomycin (0.1 mg/ml), penicillin (100 U/ml), Sodium pyruvate (1 mM; Sigma Aldrich), HEPES (10 mM; Sigma Aldrich). and L‐glutamine (2 mM; Sigma Aldrich). All viruses were packaged for 72 h following transfection of HEK‐293T cells using X‐tremeGENE 9 DNA Transfection Reagent (Roche).

### *In vivo* tumor experiments

Eight‐week‐old female nude mice (BALB/cAnNRj‐Foxn1^nu/nu^, Janvier Labs) were injected subcutaneously with 2.0 × 10^6^ (JUNB experiment) or 2.5 × 10^6^ (shT28 experiments) A375 cells in 100 μl of Matrigel (#354263, Corning) diluted to 50% in PBS. Tumor size was measured every 3^rd^ day using a digital caliper, and tumor size was calculated using the formula V = (L × W × W)/2. After the termination of the xenograft experiments, the tumors were weighed and biopsied for RNA extraction. For lung colonization experiments, 6‐ to 8‐week‐old female nude mice (BALB/cAnNRj‐Foxn1^nu/nu^, Janvier Labs) were injected intravenously (tail vein) with 1.5 × 10^5^ A375‐MA2 cells in 100 μl PBS. The animals were sacrificed after 8 weeks, and lungs were collected and fixed for 48 h in 4% paraformaldehyde before being analyzed for lung tumors in a dissecting microscope. Lung tumor counts were performed in a blinded manner. Eight‐week‐old female C57BL6/J were injected subcutaneously with 1 × 10^5^ B16.F10 cells in 200 μl of Matrigel (#354263, Corning) diluted to 50% in PBS. After the termination of the experiments on day 14, the tumors were weighed and biopsied for RNA extraction. Mice were housed in a specific pathogen‐free animal facility at the Center for Molecular Medicine, Karolinska Institutet. The study was approved by the Ethical Review Committee North, Stockholm County (Ethical approval Dnr 7885‐2017), and animals were handled in compliance with the guidelines at Karolinska Institutet.

### Matrigel invasion assay

A 2.5 × 10^4^ A375, A2058, or SK‐MEL‐28 cells were seeded in 0.1% FBS DMEM in 24‐well Matrigel GFR Invasion Chambers (#734‐1049, Corning). The inlets were put in wells containing 10% FBS DMEM and incubated for 24 h. The cells were fixed in buffered 4% formalin, permeabilized with 100% methanol, and stained using Crystal Violet. For each inlet, five images were obtained randomly using a Nikon TMS‐F microscope with a DeltaPix camera module, and cells were counted and averaged for each inlet using ImageJ.

### TRIM28 interactome analysis

A375 cells were transduced with pBABE‐BioID2‐TRIM28 or pBABE‐BioID2 retrovirus followed by selection with 1 μg/ml puromycin. Transduced cells were then cultured in the presence of 50 μM Biotin (Sigma Aldrich) for 20 h prior to lysis and then bound to Dynabeads MyOne Streptavidin C1 (Thermo Fisher Scientific) magnetic beads overnight. We then performed streptavidin pull‐down of biotinylated proteins following the procedure described by Roux *et al* (2018). After enrichment of biotinylated proteins, they were on‐bead digested using trypsin. Peptides were dissolved in 25 μl of 2% acetonitrile and 0.1% formic acid. Ten percent of the sample was analyzed by nano‐LC‐MS/MS using an Easy‐1000 nLC chromatographic system (Thermo Fisher Scientific) coupled to a Q Exactive Plus mass spectrometer (Thermo Fisher Scientific). The peptides were separated using a heated (55°C) 50 cm C‐18 Easy‐column (Thermo Fisher Scientific), and the separation was performed using an acetonitrile/water gradient (buffer A: 2% acetonitrile, 0.1% formic acid; buffer B: 98% acetonitrile, 0.1% formic acid) of 4–26% B over 120 min, followed by a 26–95% B over 5 min and 95% B for 8 min. The flow rate was 300 nl/min. The instrument was operated in a data‐dependent mode selecting the 16 most intense precursors in the survey mass scans at 140,000 and followed by MS/MS data acquisition at 70,000 mass resolution using higher‐energy collisional dissociation (HCD) fragmentation. To discriminate genuine interactors from contaminating proteins and non‐specifically bound proteins, we filtered identified proteins using the CRAPome contamination repository (http://crapome.org/). Network analysis of identified proteins was then performed using Ingenuity Pathway Analysis software (Qiagen).

### Plasmid constructs

To express short‐hairpin RNA (shRNA), we used the following lentiviral plasmids: pLKO.1‐TRIM28‐1 (TRCN0000018001), pLKO.1‐TRIM28‐2 (TRCN0000018002), pLKO.1‐TRIM28‐3 (TRCN0000017998), pLKO.1‐Trim28 (TRCN0000071366), and pLKO.1‐YAP1 (TRCN0000107266). As non‐targeting controls, we used pLKO.1‐encoding scrambled (shSCR) or luciferase (shLUC)‐specific shRNA. For CRISPRi, we inserted gRNA sequences into pLV‐hU6‐sgRNA‐hUbC‐dCas9‐KRAB‐T2a‐Puro (#71236, Addgene). The following gRNA sequences were used for CRISPRi: gJUNB‐1 TAGCGCGGTATAAAGGCGTG, gJUNB‐2 CCAATCGGAGCGCACTTCCG, and gEGFP GACCAGGATGGGCACCACCC. To generate JUNB‐expressing retrovirus, FLAG‐JUNB was excised from pCS2‐FLAG‐JUNB (#29687, Addgene) and inserted into the pBABE‐puro backbone. To generate FOSL1‐expressing retrovirus, the full coding sequence of FOSL1 was cloned by PCR and inserted into pFLAG‐CMV‐6c, followed by transfer of the FLAG‐FOSL1 fragment into the pBABE‐puro backbone. To identify the TRIM28 interactome in melanoma cells, the coding sequence for TRIM28 was inserted into the pBABE‐BioID2 plasmid (#80900, Addgene). To diminish the risk for steric hindrance, and to increase the radius of the interactome, a flexible linker (GGGGS) was inserted between the BioID2 tag and TRIM28. All lentiviruses were packaged using pMD2.G (#12259, Addgene) and psPAX2 (#12260, Addgene). Retroviruses were packaged using pMD2.G (#12259, Addgene) and pUMVC (#8449, Addgene).

### Quantitative PCR

All RNA extractions were performed using TRIzol (Invitrogen). cDNA conversions were performed using the High‐Capacity cDNA Reverse Transcription kit (Applied Biosystems) or the iScript cDNA Synthesis kit (Bio‐Rad). Gene expression was determined using TaqMan Gene Expression Assays (Thermo Fisher Scientific): *TRIM28* (Hs00232212_m1), *Trim28* (Mm00495594_m1), *ANKRD1* (Hs00173317_m1), *CYR61* (Hs00998500_g1), *CTGF* (Hs00170014_m1), *FOSL1* (Hs00606343_g1), *JUNB* (Hs00357891_s1), *CXCL1* (Hs00236937_m1), *CXCL2* (Hs00601975_m1), *CXCL8* (Hs00174103_m1), *TGFB2* (Hs00234244_m1), *YAP1* (Hs00902712_g1), *HPRT1* (Hs01003267_m1), *Hprt1* (Mm01545399_m1), *ACTB* (Hs01060665_g1), *UBC* (Hs00824723_m1), and *Gapdh* (Mm00484668_m1). Precipitated genomic sequences after ChIP in RNAPII pausing experiments were detected using SYBR‐based quantitative PCR (iQ SYBR Green Supermix, Bio‐Rad) using the following primers: JUNB‐TSS (F: GGCTGGGACCTTGAGAGC, R: GTGCGCAAAAGCCCTGTC) and JUNB‐1 kb (F: CATCAAAGTGGAGCGCAAG, R: TTGAGCGTCTTCACCTTGTC).

### Inhibitors

We used the following inhibitors: Puromycin (Thermo Fisher Scientific) and Halt™ Protease and Phosphatase Inhibitor Cocktail (Thermo Fisher Scientific).

### Antibodies for immunoblotting and immunofluorescence

Cell lysates for immunoblotting were prepared using CelLytic M (Sigma Aldrich) supplemented with a protease and phosphatase inhibitor cocktail and separated on 4–20% Mini‐PROTEAN TGX Precast Protein Gels (Bio‐Rad). Proteins were transferred to an Amersham Hybond PVDF membrane (GE Healthcare) using semi‐dry transfer, and the binding of HRP‐conjugated antibodies was visualized using Clarity Western ECL Substrate (Bio‐Rad). For immunoblotting, we used the following antibodies: anti‐β‐actin‐HRP clone AC‐15 (A3854, Sigma Aldrich), anti‐HSP90‐HRP (sc‐13119, Santa Cruz Biotechnology), anti‐TRIM28 (ab10484, Abcam), anti‐JUNB (#3753, Cell Signaling Technologies), anti‐YAP1 (#4912, Cell Signaling Technologies), anti‐FRA1 (#5281, Cell Signaling Technologies), anti‐phospho‐YAP1 (#4911, Cell Signaling Technologies), anti‐YAP/TAZ (#8418, Cell Signaling Technologies), anti‐ERK1/2 (#9102, Cell Signaling Technologies), anti‐phospho‐ERK1/2 (#9106, Cell Signaling Technologies), anti‐pan‐TEAD (#13295, Cell Signaling Technologies), and anti‐RNA polymerase II (ab817, Abcam). The following secondary antibodies were used: anti‐mouse IgG‐HRP (#7076, Cell Signaling Technologies), conformation‐specific anti‐rabbit IgG‐HRP (#5127, Cell Signaling Technologies), and anti‐rabbit IgG‐HRP (P0448, Agilent Dako). All antibodies were used as recommended by the manufacturers. ImageJ was used for densitometry https://imagej.nih.gov/ij/. For immunofluorescence, the cells were fixed in 4% formalin for 15 min at room temperature followed by permeabilization in 0.2% Triton‐X‐100 and blocking in 5% goat serum. Cells were stained with anti‐YAP1 (#4912, Cell Signaling Technologies) or anti‐TRIM28 (ab10484, Abcam) followed by washing and staining with the secondary antibody anti‐rabbit‐IgG‐488 made in goat (Thermo Scientific). A ZOE Fluorescent Imager (Bio‐Rad) was used for taking pictures of stained cells.

### Phospho‐MAPK array

The phosphorylation of signaling mediators was analyzed using the Human Phospho‐MAPK Antibody Array (#ARY002B, R&D Systems) according to the manufacturer's protocol. Protein lysates were used from A375 and A2058 cells transduced with shSCR or shT28‐1 lentivirus. The HRP‐coupled streptavidin from the kit was replaced with IRdye 800CW Streptavidin (LI‐COR Biosciences), and all signals were analyzed using an Odyssey CLx Imaging System (LI‐COR Biosciences).

### Co‐immunoprecipitation

Approximately 2 × 10^7^ A375 cells were lysed in 1 ml of ice‐cold lysis buffer (25 mM Tris–HCl pH 7.4, 150 mM NaCl, 1% NP‐40, 1 mM EDTA, and 5% glycerol) supplemented with a protease and phosphatase inhibitor cocktail. The lysate was rotated slowly at 4°C for 30 min. After pre‐clearing of the lysate, 1 μg anti‐JUNB (#3753, Cell Signaling Technologies) or 1 μg isotype control antibody (#3900, Cell Signaling Technologies) was added to 1.0 mg protein lysate, followed by slow rotation at 4°C for 16 h. 1.5 mg of magnetic Dynabeads Protein G (Thermo Fisher Scientific) was then added to each lysate, followed by slow rotation at 4°C for 4 h. Beads were washed three times in ice‐cold washing buffer (10 mM Tris–HCl pH 7.4, 150 mM NaCl, 1 mM EDTA, and 0.1% Triton X‐100) supplemented with a protease and phosphatase inhibitor cocktail. Elution was performed by incubating the beads with SDS–PAGE sample buffer at 50°C for 10 min. Eluates were separated by SDS–PAGE before detection by immunoblotting.

### Gene perturbation with CRISPR interference and RNAi

A375, A2058, and SK‐MEL‐28 cells were transduced with lentiviruses (LKO.1) that expressed non‐targeting control shRNA (shSCR or shLuc) or shRNA specific for TRIM28 (shT28‐1 or shT28‐2) or YAP1 (shYAP1). After selection in puromycin (1 μg/ml), knockdown efficiency was validated by quantitative RT–PCR (qRT–PCR) and immunoblotting. For CRISPRi, A375 cells were transduced with lentivirus‐encoding dCas9‐KRAB and gRNAs specific for JUNB or EGFP. After selection in puromycin (1 μg/ml), the reduction in expression levels was verified using quantitative RT–PCR and immunoblotting. For siRNA‐mediated knockdown of JUNB, A375 cells were transfected with siRNA pools targeting JUNB (#L‐003269‐00‐0005, Dharmacon) or non‐targeting control siRNA (#D‐001810‐10‐05, Dharmacon), and all cells were analyzed 72 h post‐transfection. For siRNA‐mediated knockdown of YAP1 and TAZ, A375 cells were transfected with siRNA against YAP1 (ID#:107951 and ID#:107952, Ambion), TAZ (ID#:122501 and ID#:122502, Ambion), or non‐targeting siRNA (Catalog#: AM4641, Ambion), and Matrigel assays were performed 72 h post‐transfection. All siRNA transfections were performed using Lipofectamine RNAiMAX (Thermo Fisher Scientific).

### Statistical analysis

Statistical analyses were performed using R (R 3.4.4) or Prism (GraphPad Software). The specific statistical tests used are described in the figure legends.

## Author contributions

**William A Nyberg:** Conceptualization; data curation; formal analysis; supervision; validation; visualization; methodology; writing—original draft; project administration; writing—review and editing. **Diego A Velasquez Pulgarin:** Data curation; formal analysis; validation; methodology; writing—review and editing. **Tianlin He:** Data curation; formal analysis; validation; methodology. **Maria Sjöstrand:** Formal analysis; validation; methodology. **Lucia Pelle:** Formal analysis; methodology. **Ruxandra Covacu:** Formal analysis; visualization; writing—review and editing. **Alexander Espinosa:** Conceptualization; data curation; formal analysis; supervision; funding acquisition; investigation; visualization; writing—original draft; project administration; writing—review and editing.

## Disclosure and competing interests statement

The authors declare that they have no conflict of interest.

## Supporting information



Expanded View Figures PDF

Source Data for Expanded View

PDF+

Source Data for Figure 3

Source Data for Figure 4

## Data Availability

The datasets produced in this study are available in the following databases: RNA‐seq and CUT&RUN data: Gene Expression Omnibus GSE210579 (https://www.ncbi.nlm.nih.gov/geo/query/acc.cgi?acc=GSE210579). Microarray data: Gene Expression Omnibus GSE133073 (https://www.ncbi.nlm.nih.gov/geo/query/acc.cgi?acc=GSE133073).
