# Supplementary material for: The bromodomain protein TRIM28 controls the balance between growth and invasiveness in melanoma
Source: EMBO Rep. 2022 Nov 7;24(1):e54944. doi: 10.15252/embr.202254944 (PMC9827549; doi:10.15252/embr.202254944)
Supplement: Supplementary file 1 — Expanded View Figures PDF [file EMBR-24-e54944-s002.pdf]

Expanded View Figures

Figure EV1. TRIM28 is associated with poor survival, increased expression of metastatic gene signatures, and the invasiveness of melanoma cells.

A Univariate Cox regression analysis of bromodomain gene expression and overall survival in metastatic melanoma. SKCM data (TCGA) were analyzed for bromodomain gene expression in metastases ($n = 367$) and the overall survival of corresponding patients.
B Validation of TRIM28 knockdown using immunoblotting on cell lysates from A375 cells transduced with shLUC, shSCR, shT28-1, or shT28-2 lentiviruses.
C Densitometry (relative to β-actin) of the immunoblot in (B).
D GSEA of gene signatures associated with metastasis of melanoma to distant organs (KAUFFMANN_MELANOMA_RELAPSE_UP or WINNEPENNINCKX_MELANOMA_METASTASIS_UP) in metastases ($n = 367$; *left*) with high (T28hi) or low (T28lo) TRIM28 expression, and in A375 cells (*right*) transduced with two shNTC (shSCR and shLUC) or shT28-1 and shT28-2 ($n = 3$ per construct). RNA-seq data from melanoma metastases were downloaded from The Cancer Genome Atlas (TCGA).
E Eight-week-old female nude mice were injected subcutaneously with 2.5×10^6 A375 cells transduced with shSCR or shT28-3 lentivirus. Tumor size was measured every 3rd day and tumor size was calculated using the formula $V = (L \times W \times W)/2$ ($n = 10$ mice per group). Results are expressed as mean ± SEM. Statistics were calculated using repeated measures ANOVA.
F Tumor weight analyzed 17 days after subcutaneous injection of cells transduced with shSCR or shT28-3 lentivirus ($n = 10$ mice per group). Results are expressed as mean ± SEM. Statistics were calculated using the two-sided Mann–Whitney *U*-test.
G Eight-week-old female C57BL6/J were injected subcutaneously with 1×10^5 B16.F10 cells transduced with LKO.1 lentivirus targeting Firefly luciferase (shLuc; $n = 5$) or Trim28 (shT28; $n = 4$). Tumor weight was measured after 14 days, and statistics were calculated using the two-sided Mann–Whitney *U*-test. Results are expressed as mean ± SEM.
H Validation of TRIM28 knockdown in A375 cells using shT28-3 (top panel) and in B16.F10 using an shRNA specific for mouse *Trim28* (bottom panel).
I Reduced expression of the YAP1 target genes *CTGF* and *CYR61* in A375-MA2 cells after TRIM28 knockdown. One-way ANOVA and Dunnett's *post hoc* test were used for statistical testing.

Data information: *P*-values in (E, F, G, and I): $^*P < 0.05$, $^{**}P < 0.01$, $^{***}P < 0.001$.
Source data are available online for this figure.

© 2022 The Authors Published under the terms of the CC BY 4.0 license

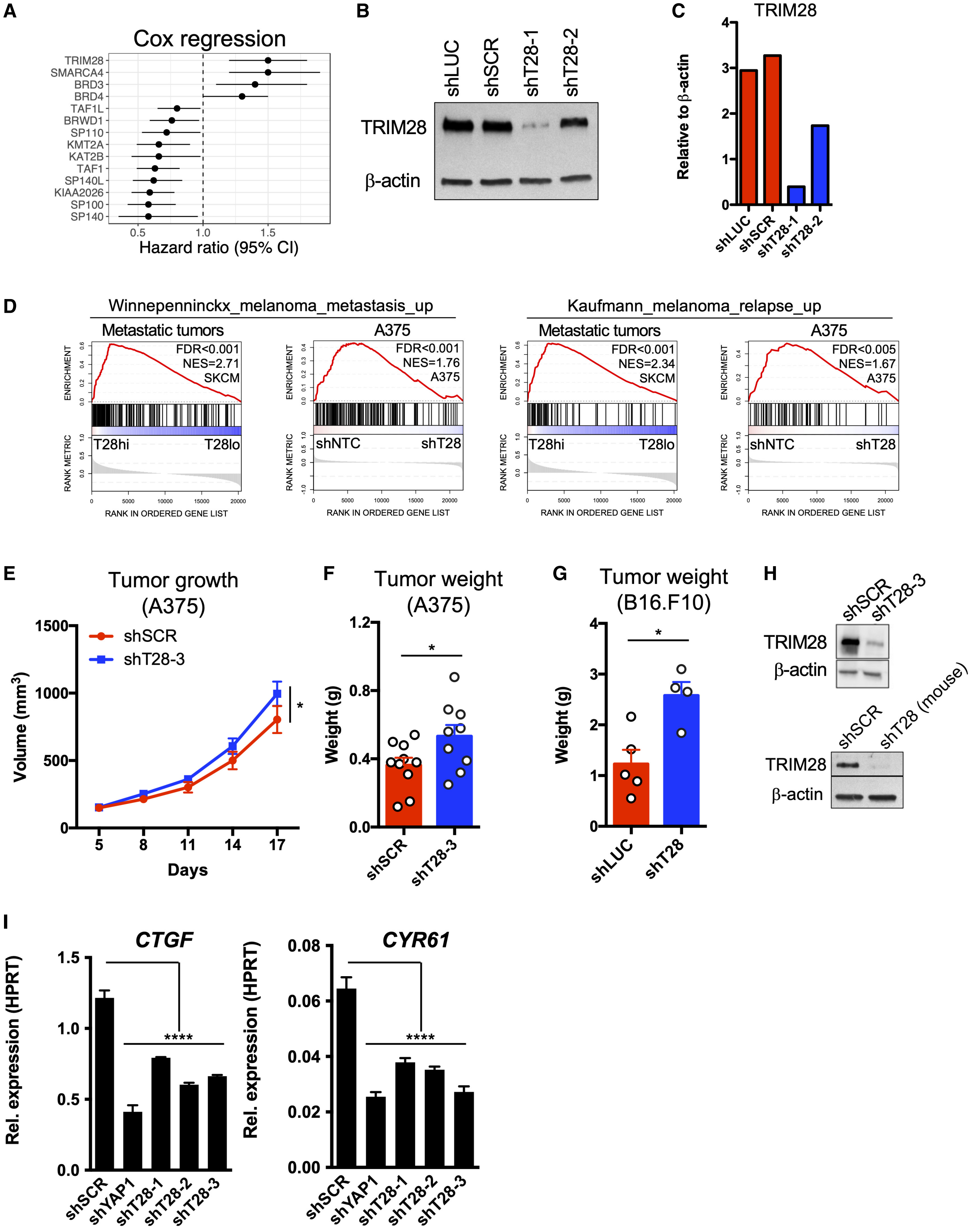

Figure EV1.

 © 2022 The Authors Published under the terms of the CC BY 4.0 license

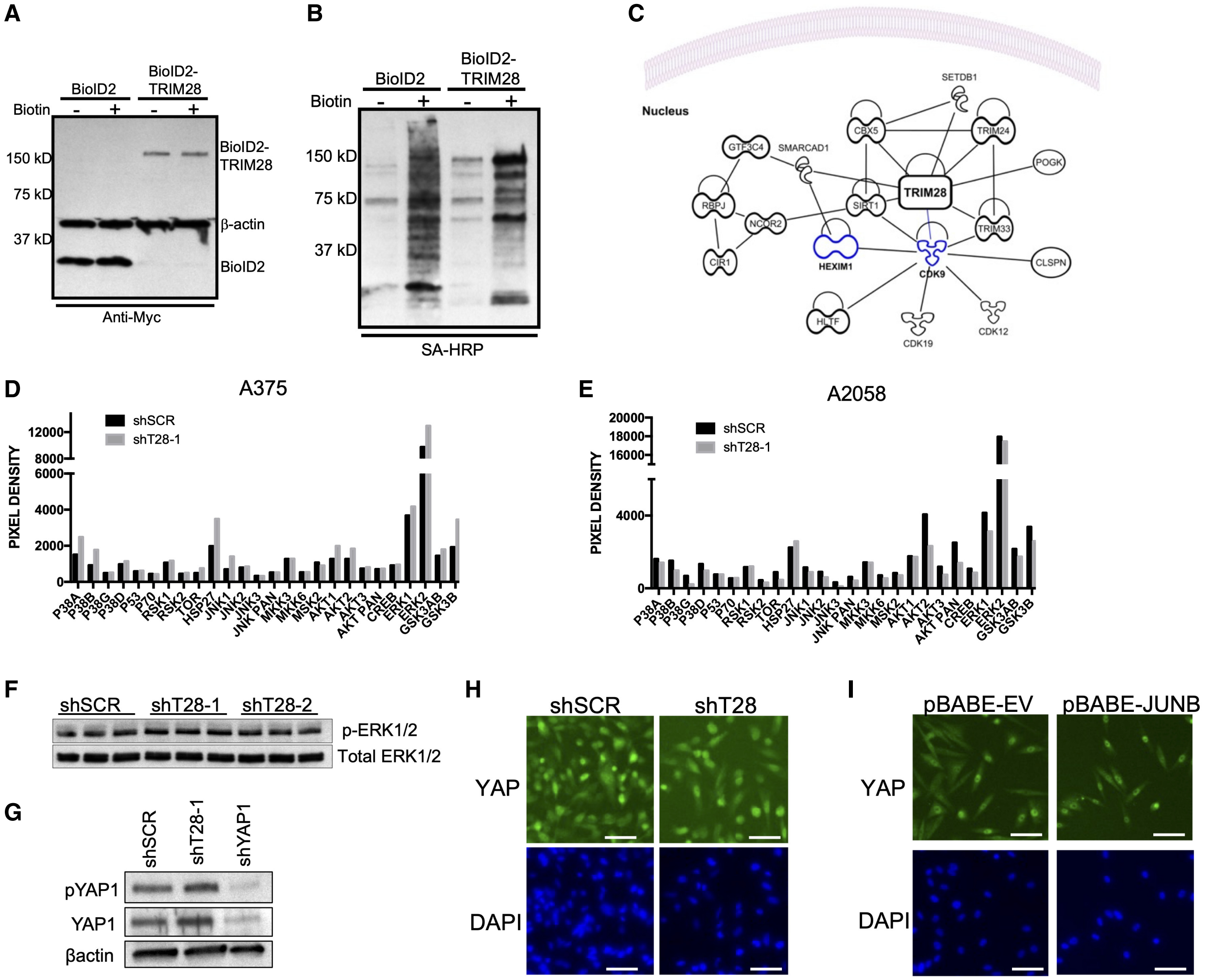

Figure EV2. TRIM28 interacts with epigenetic regulators and factors regulating transcriptional elongation and does not directly affect ERK or YAP signaling.

A A375 cells were transduced with pBABE-BioID2-TRIM28 or pBABE-BioID2 followed by selection with 1 μg/ml puromycin. Expression of BioID2 and BioID2-TRIM28 in cell lysates was verified with immunoblotting using an anti-Myc antibody.

B A375 cells were transduced with pBABE-BioID2-TRIM28 or pBABE-BioID2. Transduced cells were then cultured in the presence of 50 μM biotin for 20 h prior to lysis and then bound to Dynabeads MyOne Streptavidin C1 magnetic beads overnight. After the enrichment of biotinylated proteins, they were detected using streptavidin–HRP.

C Protein–protein interaction network based on identified TRIM28 interactors was analyzed by ingenuity pathway analysis (IPA). Displayed interactors are based on experimentally validated direct and indirect interactors. KRAB-ZFN proteins (> 50) are not displayed for increased clarity.

D A375 cells were transduced with shSCR or shT28-1 lentivirus followed by selection in a medium containing 1 μg/ml puromycin cells. Cells were lysed and lysates were applied to Human Phospho-MAPK Antibody Array.

E A2058 cells were transduced with shSCR or shT28-1 lentivirus followed by selection in a medium containing 1 μg/ml puromycin cells. Cells were lysed and lysates were applied to Human Phospho-MAPK Antibody Array.

F A375 cells were transduced with shSCR, shT28-1, or shYAP1 lentivirus. After selection, 1 μg/ml puromycin cells were lysed and proteins were separated on 4–20% SDS–PAGE. Proteins were transferred to PVDF membranes and detected with anti-ERK1/2 and anti-phospho-ERK1/2.

G A375 cells were transduced with shSCR or shT28-1 lentivirus. After selection, 1 μg/ml puromycin cells were lysed and proteins were separated on 4–20% SDS–PAGE. Proteins were transferred to PVDF membranes and detected with anti-phospho-YAP1 (pYAP1), anti-YAP1, or anti-β-actin antibodies.

H Intracellular localization of YAP1 after TRIM28 knockdown in A375 cells. Scale bar is 60 μm.

I Intracellular localization of YAP1 after JUNB overexpression in A375 cells. The scale bar is 60 μm.

Source data are available online for this figure.

© 2022 The Authors Published under the terms of the CC BY 4.0 license

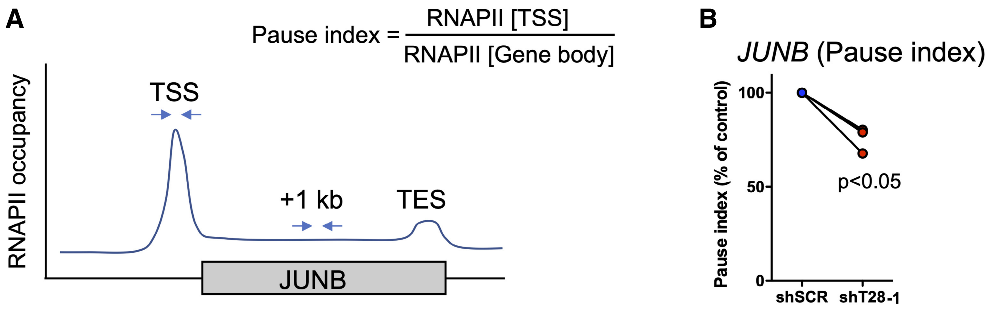

Figure EV3. ChIP-qPCR validation of reduced RNAPII pausing at the TSS of the JUNB gene.

A A375 cells were transduced with shSCR or shT28-1 lentivirus followed by selection in 1 μg/ml puromycin. After ChIP against RNAPII, qPCR was performed with primers amplifying precipitated genomic DNA from the TSS and gene body of JUNB (+1 kb).

B Results were normalized to corresponding input samples and three biological replicates are displayed as % pause index compared to shSCR control. The ratio *t*-test was used for statistical testing ($n = 3$).

Figure EV4. RNA-seq of A375 cells overexpressing JUNB or FOSL1.

A PCA plot of the different samples in the RNA-seq experiment (EV = empty vector; $n = 4$).

B Volcano plot comparing A375 cells overexpressing JUNB against A375 cells overexpressing FOSL1.

C Volcano plot comparing A375 cells overexpressing FOSL1 against A375 cells transduced with EV.

D Immunoblotting against FOSL1 after overexpression with pBABE-FOSL1 in A375 cells. HSP90 was used as a loading control.

E GSEA analysis of A375 cells overexpressing JUNB compared to cells overexpressing FOSL1 ($n = 4$).

F Validation of JUNB overexpression effect on YAP1 target genes *ANKRD1*, *CTGF*, *CYR61*, as well as *CXCL8* and *CXCL2* in A2058 cells. qRT–PCR was used to determine expression levels and results are expressed as mean ± SEM from three biological replicates ($n = 3$). Two-sided unpaired *t*-tests were used for statistical testing.

G Rescue experiment to determine the expression of typical YAP1 and RAS signature genes after siRNA (siNTC or siJUNB) transfection of A375 cells transduced with shSCR or shT28-1. qRT–PCR was used to determine expression levels relative to *UBC*, and the results are expressed as mean ± SEM from three biological replicates ($n = 3$). Two-sided unpaired *t*-tests were used for statistical testing.

H A375 cell lysate was used for immunoprecipitation of endogenous JUNB followed by detection by immunoblotting using anti-YAP1, anti-pan-TEAD, and anti-FOSL1. An isotype IgG antibody was used as a negative immunoprecipitation control.

Data information: *P*-values in (F and G): $^*P < 0.05$, $^{**}P < 0.01$, $^{***}P < 0.001$.
Source data are available online for this figure.

© 2022 The Authors Published under the terms of the CC BY 4.0 license

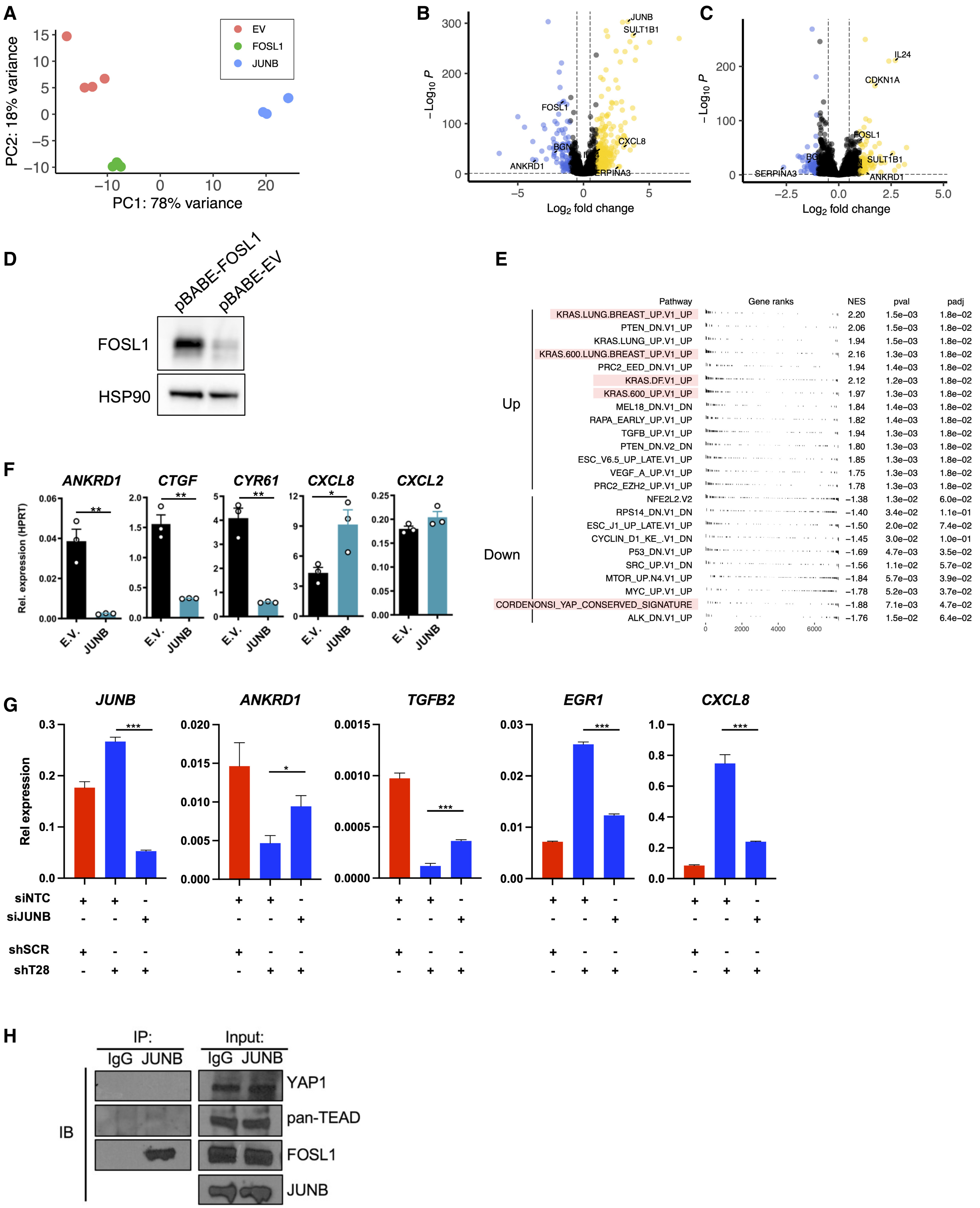
A
PC2: 18% variance
PC1: 78% variance
EV
FOSL1
JUNB
B
C
Log2 fold change
JUNB
SULT1B1
FOSL1
CXCL8
ANKRD1
IL24
CDKN1A
SERPINA3
D
pBABE-FOSL1
pBABE-EV
FOSL1
HSP90
E
Pathway
Gene ranks
NES
pval
padj
Up
Down
F
ANKRD1
CTGF
CYR61
CXCL8
CXCL2
Rel. expression (HPRT)
E.V.
JUNB
G
JUNB
ANKRD1
TGFB2
EGR1
CXCL8
Rel expression
siNTC
siJUNB
shSCR
shT28
H
IP:
Input:
IgG
JUNB
IB
YAP1
pan-TEAD
FOSL1
JUNB

Figure EV4.

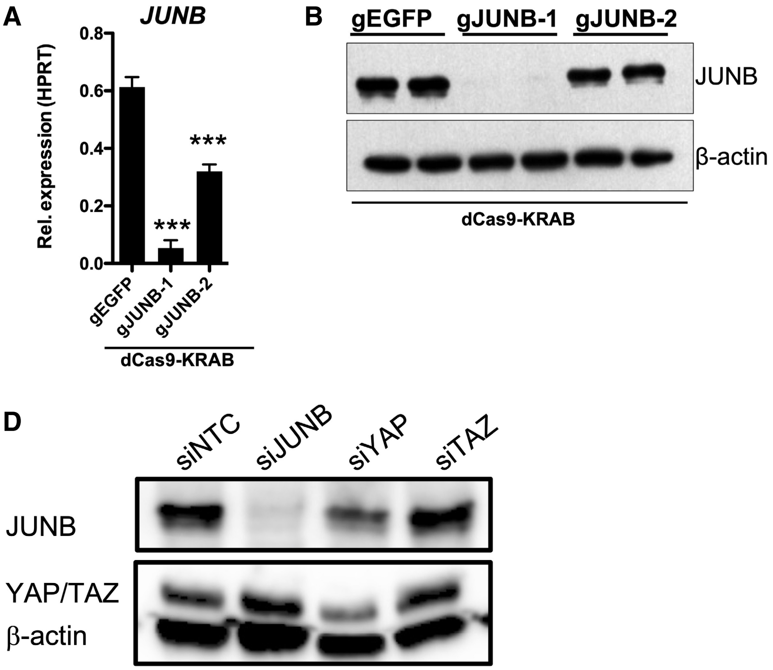

Figure EV5. Validation of JUNB, YAP, and TAZ expression after CRISPRi and siRNA.

A Expression of *JUNB* in A375 cells after transduction with dCas9-KRAB lentiviruses expressing gRNA targeting *JUNB* (gJUNB-1 or gJUNB-2) or a control gRNA (gEGFP). qRT–PCR was used to determine *JUNB* expression levels, and the results are expressed as mean ± SEM from three biological replicates ($n = 3$). One-way ANOVA and Dunnett's *post hoc* test were used for statistical testing. *$P < 0.05$, **$P < 0.01$, ***$P < 0.001$.

B Expression of JUNB in A375 cells after transduction with dCas9-KRAB lentiviruses expressing gRNA targeting *JUNB* (gJUNB-1 or gJUNB-2) or a control gRNA (gEGFP). Immunoblotting was used to determine the protein levels of JUNB. β-actin was used as loading control.

C Validation of JUNB knockdown (shown in B) by densitometry. JUNB levels are shown relative to β-actin.

D Validation of siNTC-, siJUNB-, siYAP1-, and siTAZ-mediated knockdown in A375 cells.

Source data are available online for this figure.

© 2022 The Authors Published under the terms of the CC BY 4.0 license
