# Supplementary material for: The bromodomain protein TRIM28 controls the balance between growth and invasiveness in melanoma
Source: EMBO Rep. 2022 Nov 7;24(1):e54944. doi: 10.15252/embr.202254944 (PMC9827549; doi:10.15252/embr.202254944)
Supplement: Supplementary file 2 — Source Data for Expanded View [file EMBR-24-e54944-s003.zip › EV_Figure_Source_Data/WB_Fig._EV2.pdf]

Fig. EV2A

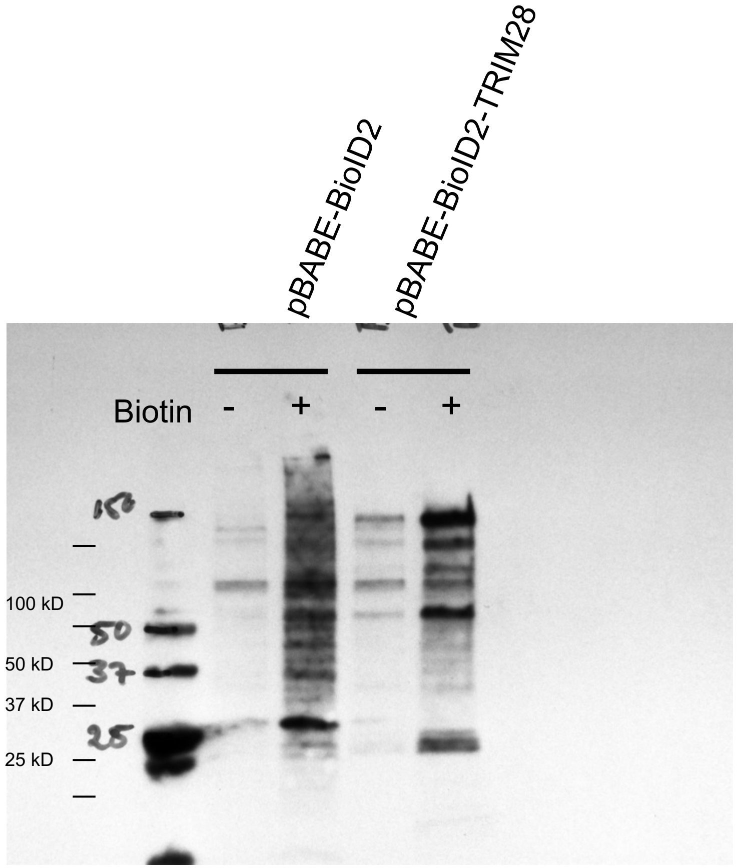

Biotinylated proteins
(Streptavidin-HRP)

Fig. EV2B

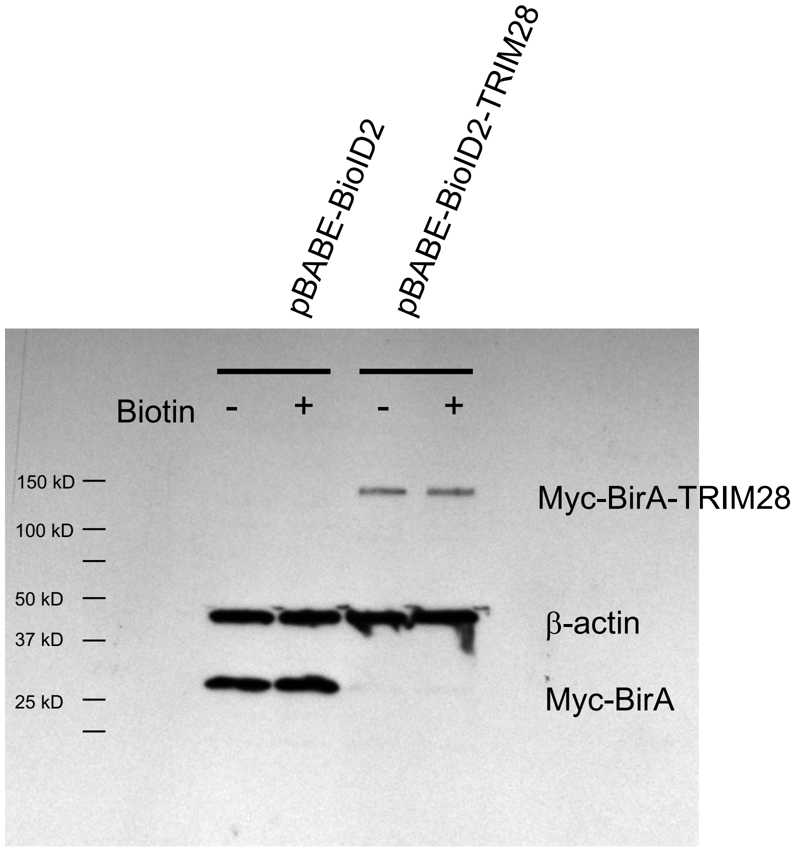

Fig. EV2F

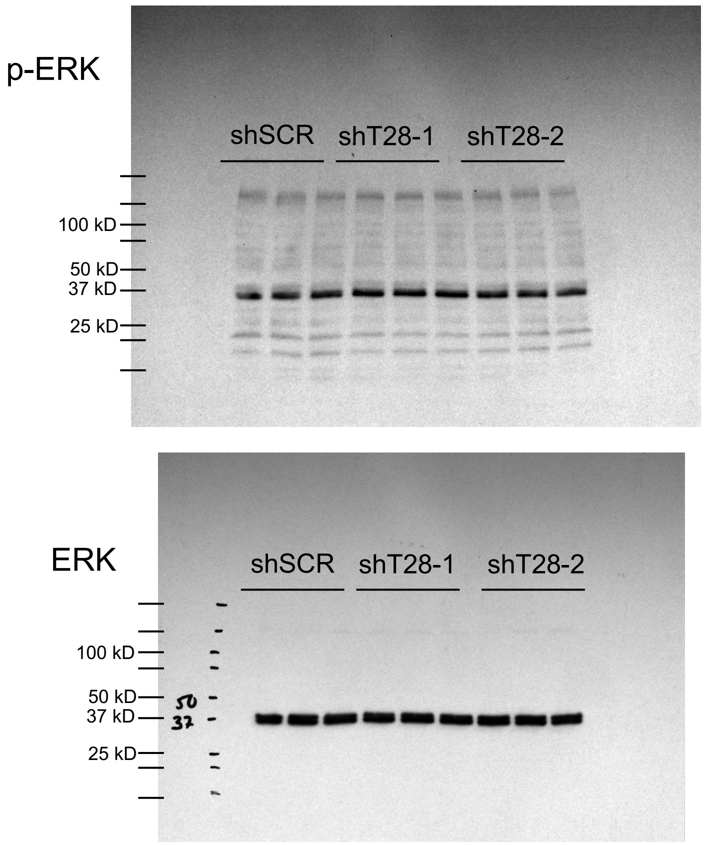

Fig. EV2G

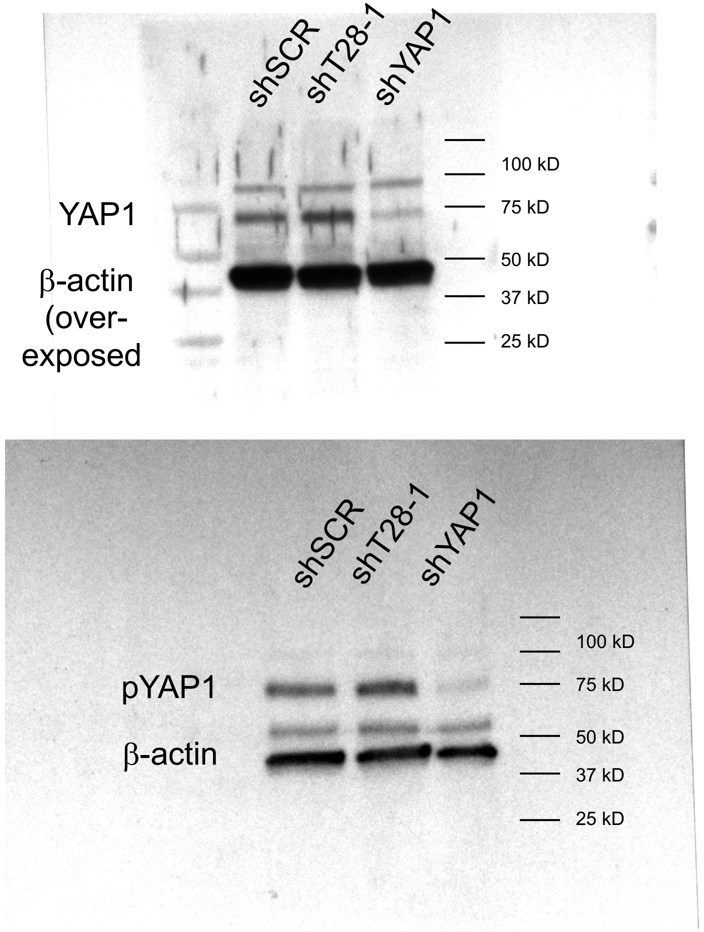

Size marker = Precision Plus Protein Dual Color (Bio-Rad)
