# Supplementary figures and images for: The bromodomain protein TRIM28 controls the balance between growth and invasiveness in melanoma
Source: EMBO Rep. 2022 Nov 7;24(1):e54944. doi: 10.15252/embr.202254944 (PMC9827549; doi:10.15252/embr.202254944)

**Fig. EV1B**

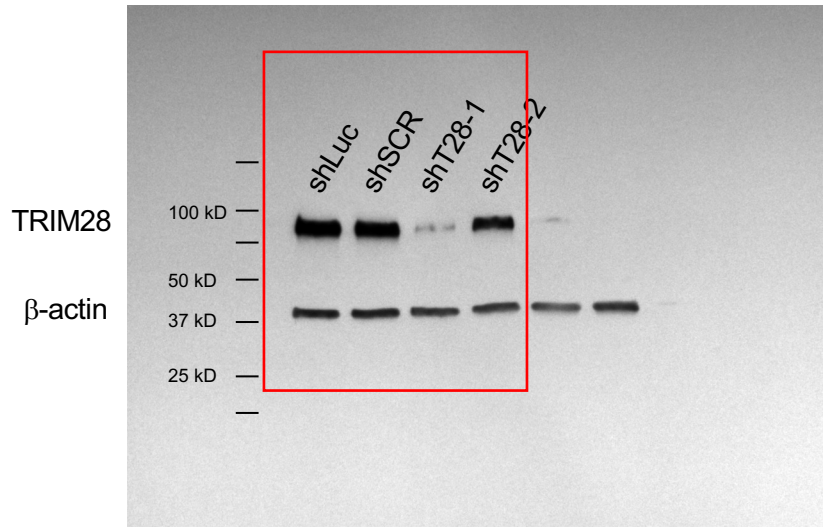

**Fig. EV1H**

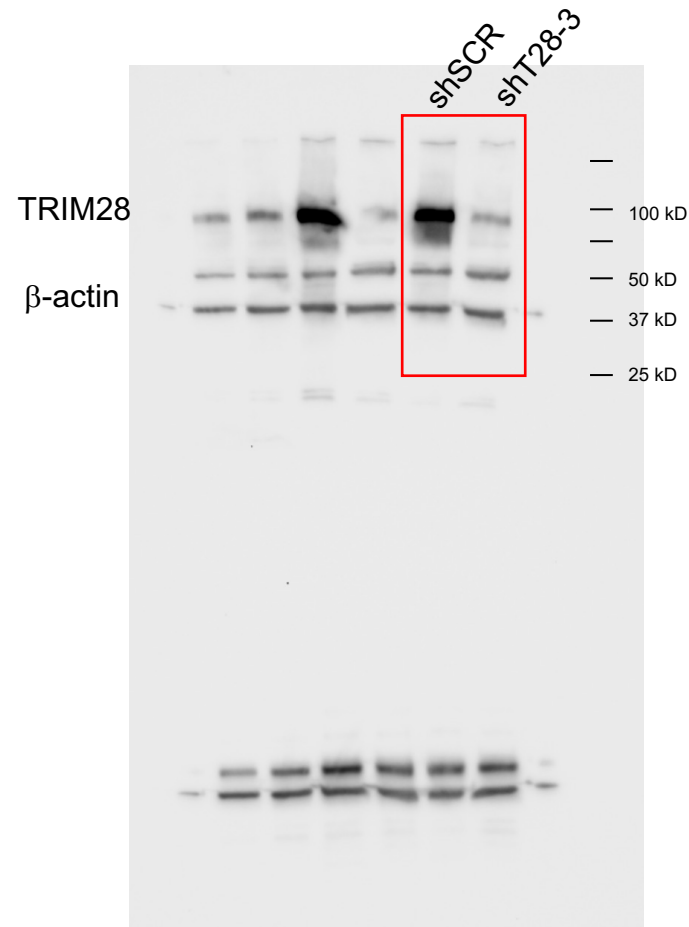

**Fig. EV1H**

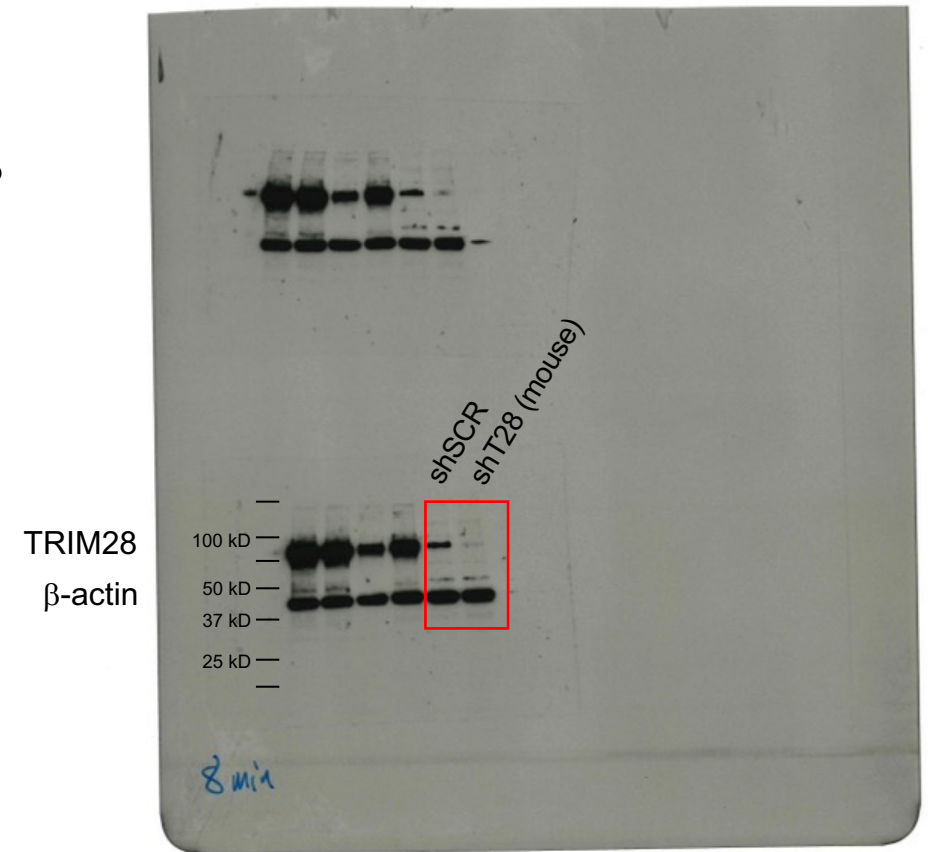

Size marker = Precision Plus Protein Dual Color (Bio-Rad)

Supplement: Supplementary file 2 — Source Data for Expanded View [file EMBR-24-e54944-s003.zip › EV_Figure_Source_Data/WB_Fig._EV1.pdf]

Fig. EV4D

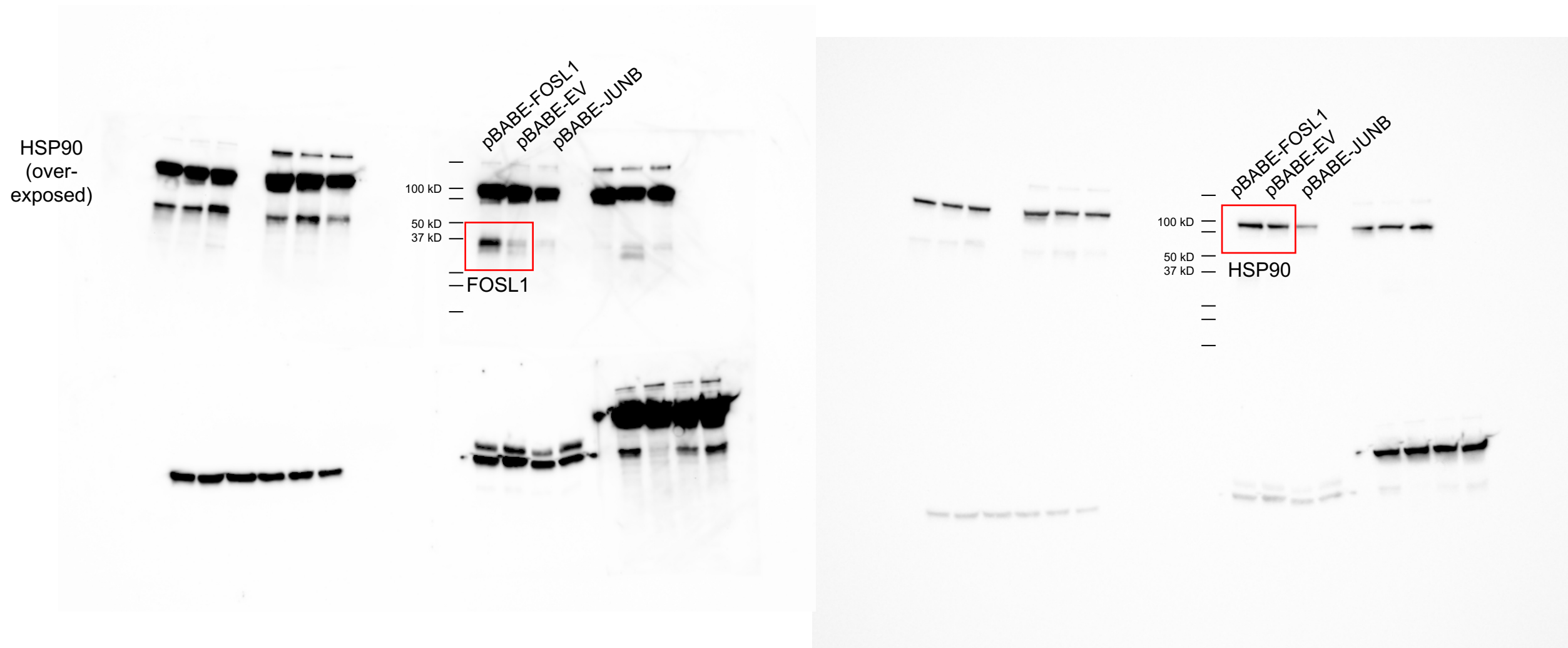

Size marker = Precision Plus Protein Dual Color (Bio-Rad)

Supplement: Supplementary file 2 — Source Data for Expanded View [file EMBR-24-e54944-s003.zip › EV_Figure_Source_Data/WB_Fig._EV4.pdf]

Fig. EV5B

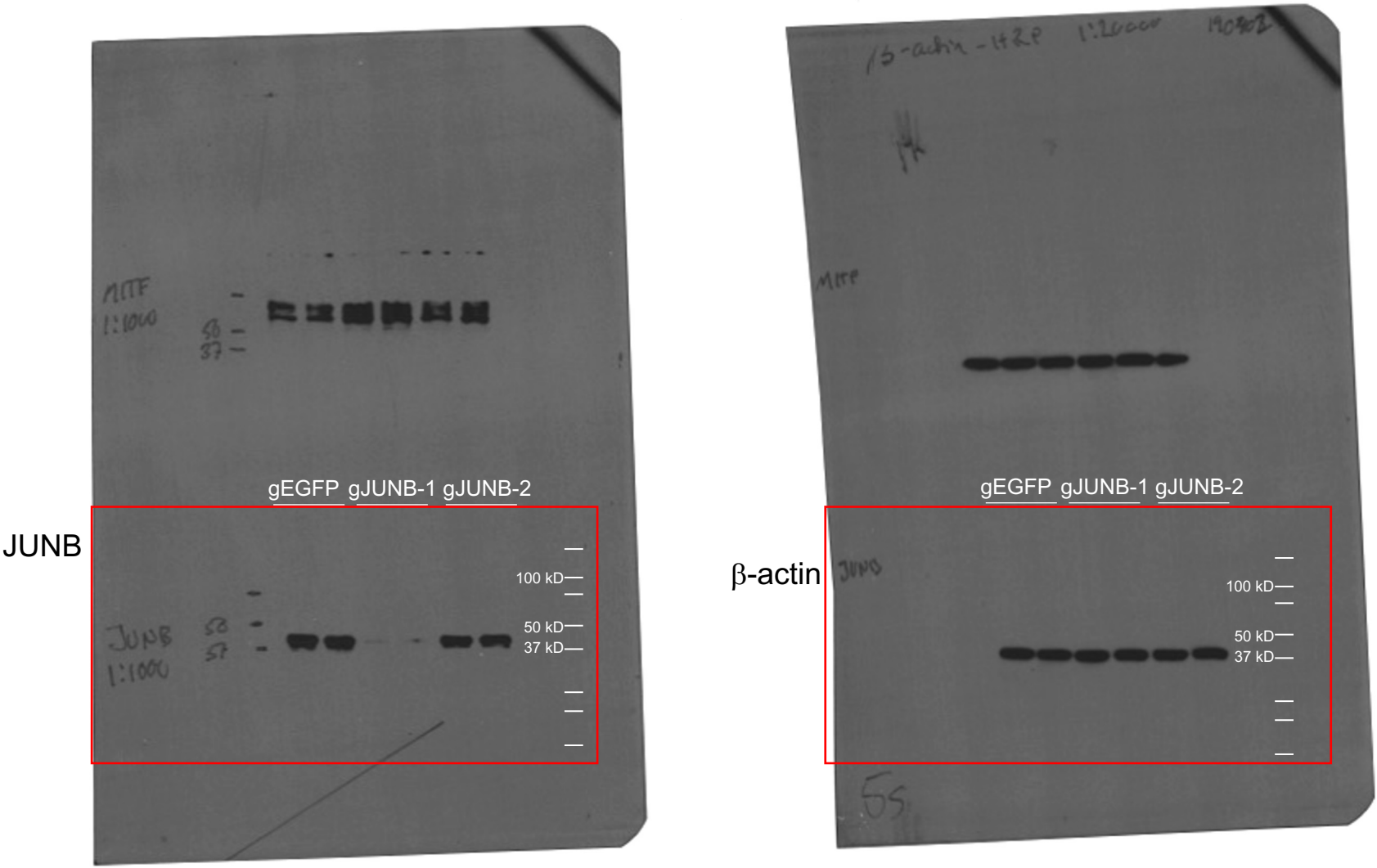

Size marker = Precision Plus Protein Dual Color (Bio-Rad)

Fig. EV5D

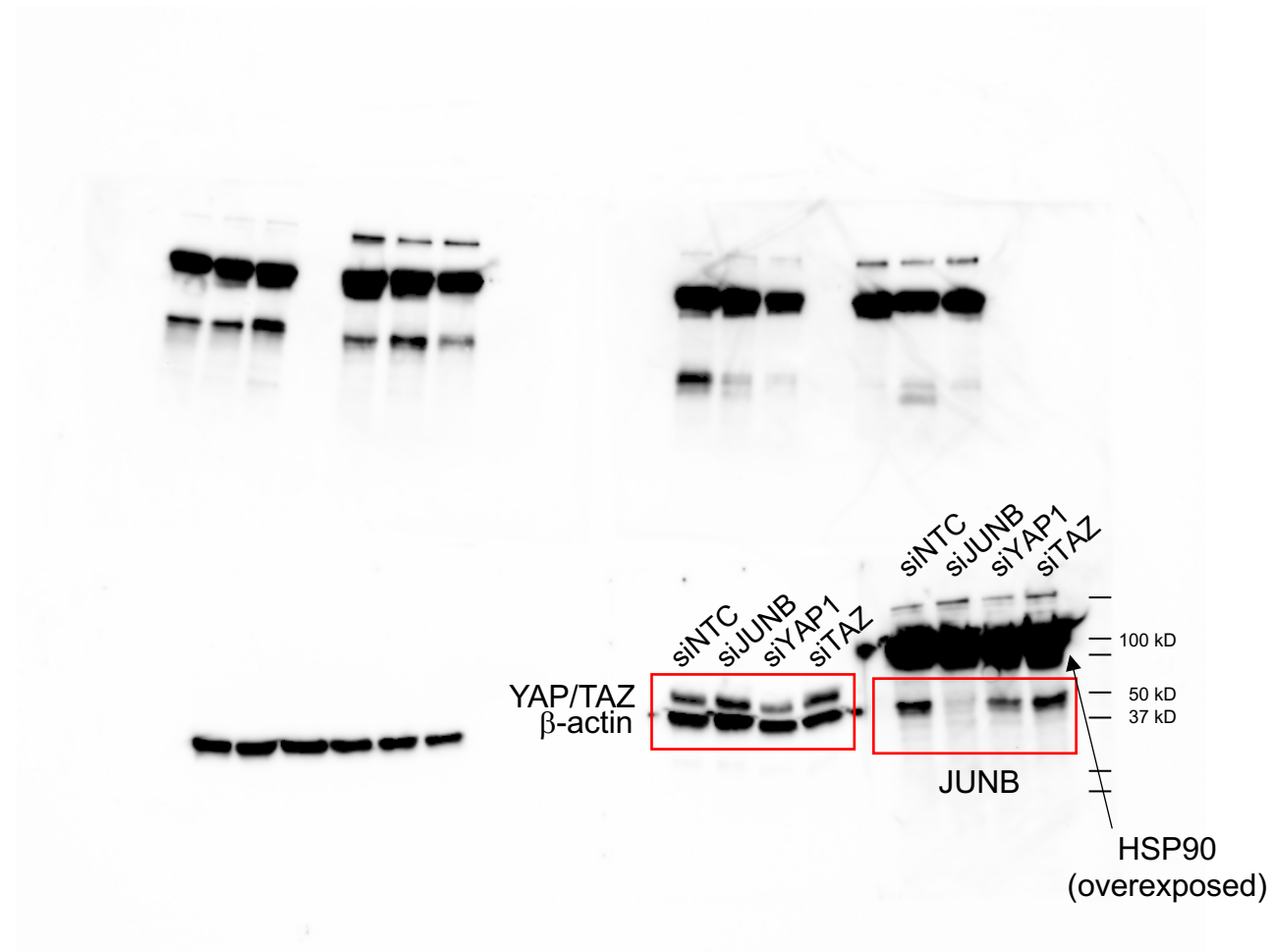

Supplement: Supplementary file 2 — Source Data for Expanded View [file EMBR-24-e54944-s003.zip › EV_Figure_Source_Data/WB_Fig._EV5.pdf]

**Fig. 3D**

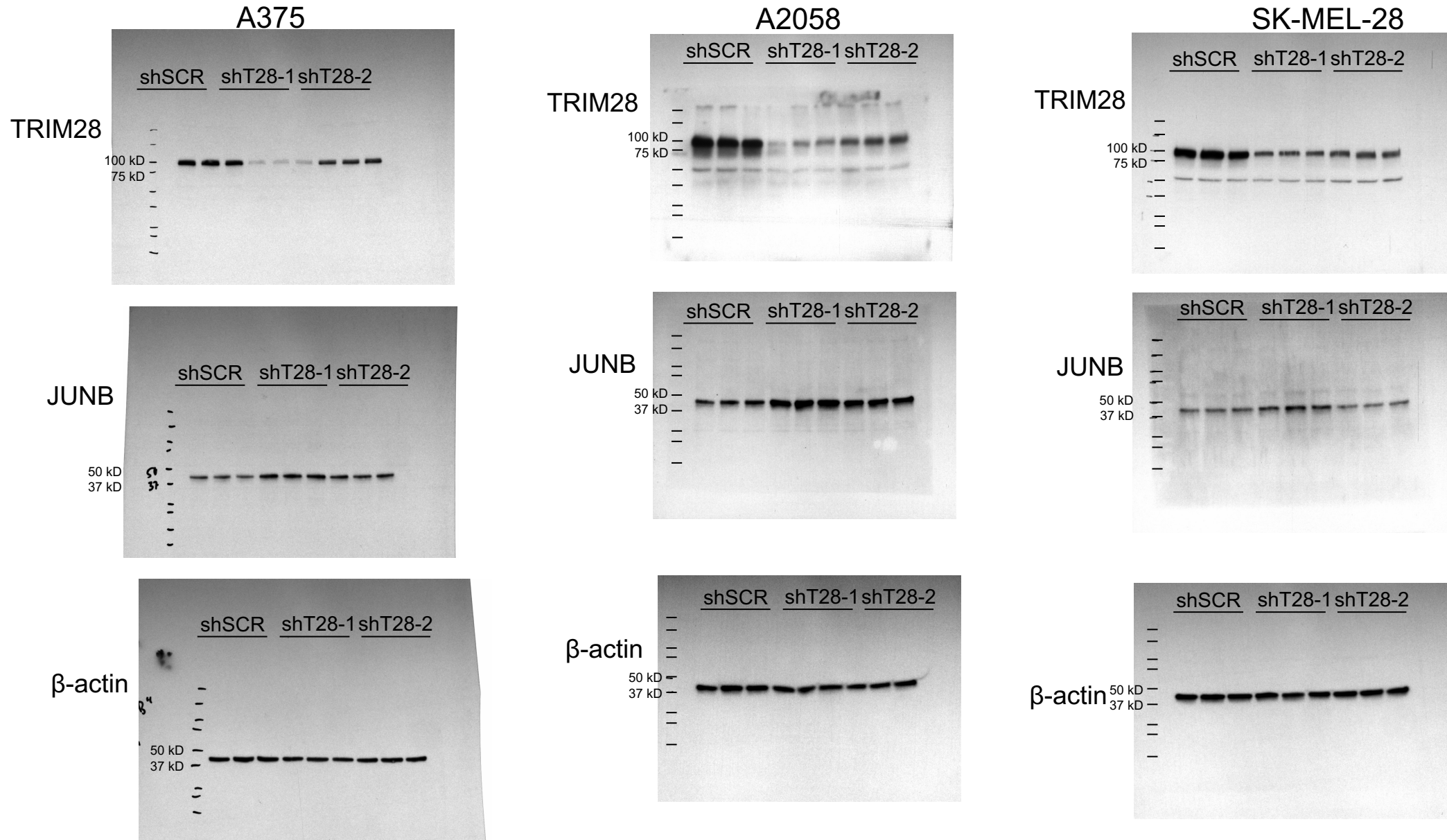

Size marker = Precision Plus Protein Dual Color (Bio-Rad)

Supplement: Supplementary file 4 — Source Data for Figure 3 [file EMBR-24-e54944-s006.pdf]

**Fig. 4A**

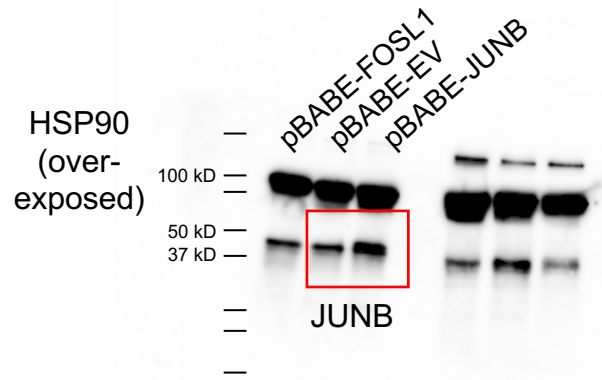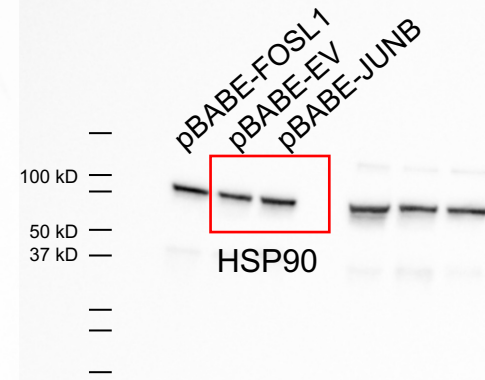

Size marker = Precision Plus Protein Dual Color (Bio-Rad)

Supplement: Supplementary file 5 — Source Data for Figure 4 [file EMBR-24-e54944-s004.pdf]
